# Synthesis of d-Galactose-Substituted Acylsilanes and Acylgermanes. Model Compounds for Visible Light Photoinitiators with Intriguing High Solubility

**DOI:** 10.1021/acs.organomet.0c00753

**Published:** 2021-04-27

**Authors:** Lukas Schuh, Philipp Müller, Ana Torvisco, Harald Stueger, Tanja M. Wrodnigg, Michael Haas

**Affiliations:** † Institute of Inorganic Chemistry, Graz University of Technology, Stremayrgasse 9, A-8010 Graz, Austria; ‡ Institute of Chemistry and Technology of Biobased Systems, Graz University of Technology, Stremayrgasse 9, A-8010 Graz, Austria

## Abstract

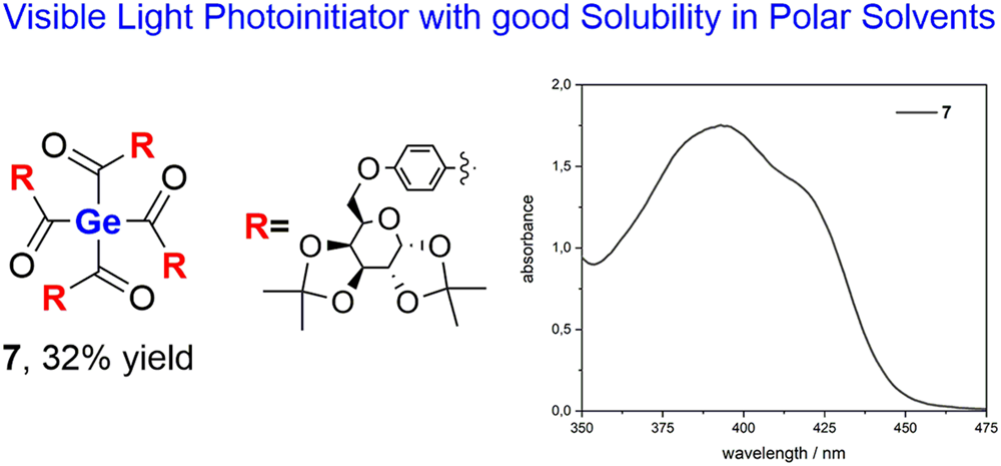

A convenient synthetic method to obtain d-galactose-substituted acylsilanes and acylgermanes is described. These acyl group 14 compounds are easily accessible in good yields. Their structural properties were analyzed by a combination of NMR, single crystal X-ray crystallography, and UV/vis spectroscopy. A d-galactose-substituted tetraacylgermane represents a new interesting visible light photoinitiator based on its absorption properties as well as its high solubility.

The demand and application of high performance photopolymers has been immensely growing in the past decades.^1^ Today, their use is no longer restricted to the manufacture of microelectronic devices, coatings, adhesives, inks, printing plates, optical waveguides but also emerging fields of medicine (dental filling materials, artificial tissue, heart valves, etc.) and fabrication of 3D objects.^2−6^ The key component in such formulations is the photoinitiator (PI). On the basis of this, their synthetic pathway is required to be sustainable, environmental friendly, and step economical.^7^ Especially for medical applications photoinitiators and their photoproducts have to be nontoxic and highly efficient. Among other promising PI systems, acylgermanes have emerged as suitable radical precursors generating acyl- and germyl-centered radicals upon irradiation, which add very rapidly to double bonds of various monomers.^8−14^ Moreover, they have the advantages of significantly red-shifted absorption bands and reduced toxicity compared to the frequently applied phosphorus-based PIs.^15−18^ The commercially available Ge-based photoinitiator [bis(4-methoxybenzoyl)diethylgermane (Ivocerin))]^11^ still suffers from inefficient curing depth at wavelengths >500 nm (compare Chart 1). Furthermore, the synthetic strategy toward Ivocerin relies on a multistep synthesis (based on the Corey–Seebach reactions), which consequently requires a complex purification process.^13^

**Chart 1:**
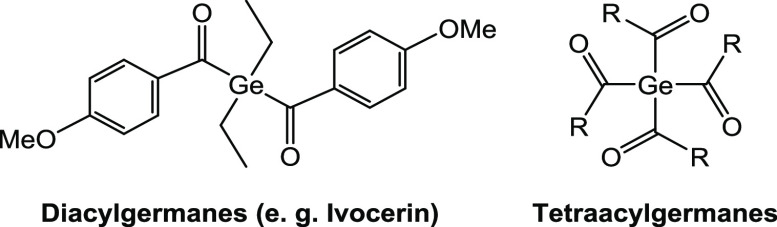
State-of-the-Art Germanium-Based Photoinitiators

Recently, we established a synthetic method to obtain tetraacylgermanes by a facile one-pot synthetic protocol (Chart 1). Their low toxicity and fast photobleaching, even upon irradiation with high-wavelength visible light, were demonstrated.^19^ Moreover, the good group tolerance of this synthetic protocol enabled us to synthesize a large variety of different substituted tetraacylgermanes. This “fine-tuning” gave us the opportunity to design photoinitiators for different applications (e.g., dental filling, 3D printing, etc.).^20^ However, a huge drawback of symmetrical tetraacylgermanes is their high melting point, which is responsible for the low solubility, limiting the field of applications.

Herein, we set out and investigated new synthetic pathways toward acyl group 14 compounds decorated with monosaccharides to induce better solubility. Moreover, the saccharides bear the possibility of subsequent deprotection, which can lead to water-soluble compounds. To prove the viability of our project, we first synthesized a monoacylsilane **1** and a monoacylgermane **2**. The acyl moiety bears a di-*O*-isopropylidene-protected galactose moiety. For both central atoms the entry into this chemistry is provided by the formation of the corresponding potassium tris(trimethylsilyl)silanide **1K** and -germanide **2K**. These anions are conveniently achievable by reaction of tetrakis(trimethylsilyl)silane and -germane with equimolar amounts of KO*t*Bu in polar solvents. Subsequently, these anions were reacted with equimolar amounts of 1,2:3,4-di-*O*-isopropylidene-α,d-galacturonic acid chloride which allows for generating the respective acyl compounds **1** and **2**, in good yields (Scheme 1).

**Scheme 1:**
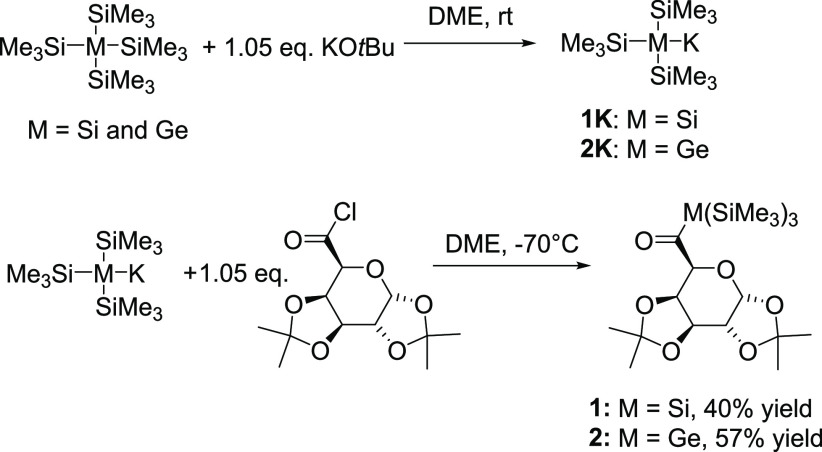
Synthesis of Monoacylsilane **1** and Monoacylgermane **2**

NMR spectra and detailed characterization for **1** and **2** are provided in the Supporting Information. Both derivatives show very similar ^13^C chemical shifts for the carbonyl C atom at 246.0 ppm (for **1**) and at 242.5 ppm (for **2**), which is characteristic for carbonyl groups directly linked to silicon or germanium atoms. The ^29^Si NMR spectra of **1** and **2** showed one resonance for the three trimethylsilyl groups at −11.20 ppm (for **1**) and −4.85 ppm (for **2**), and for compound **1** one resonance was found for the silicon atom bearing the acyl group near −70 ppm. The molecular structures of **1** and **2**, as determined by single-crystal X-ray crystallography, are depicted in Figures 1 and 2. Both compounds crystallized in the orthorhombic space group *P2*_1_2_1_2_1_ with unexceptional bond lengths and angles.

**Figure 1:**
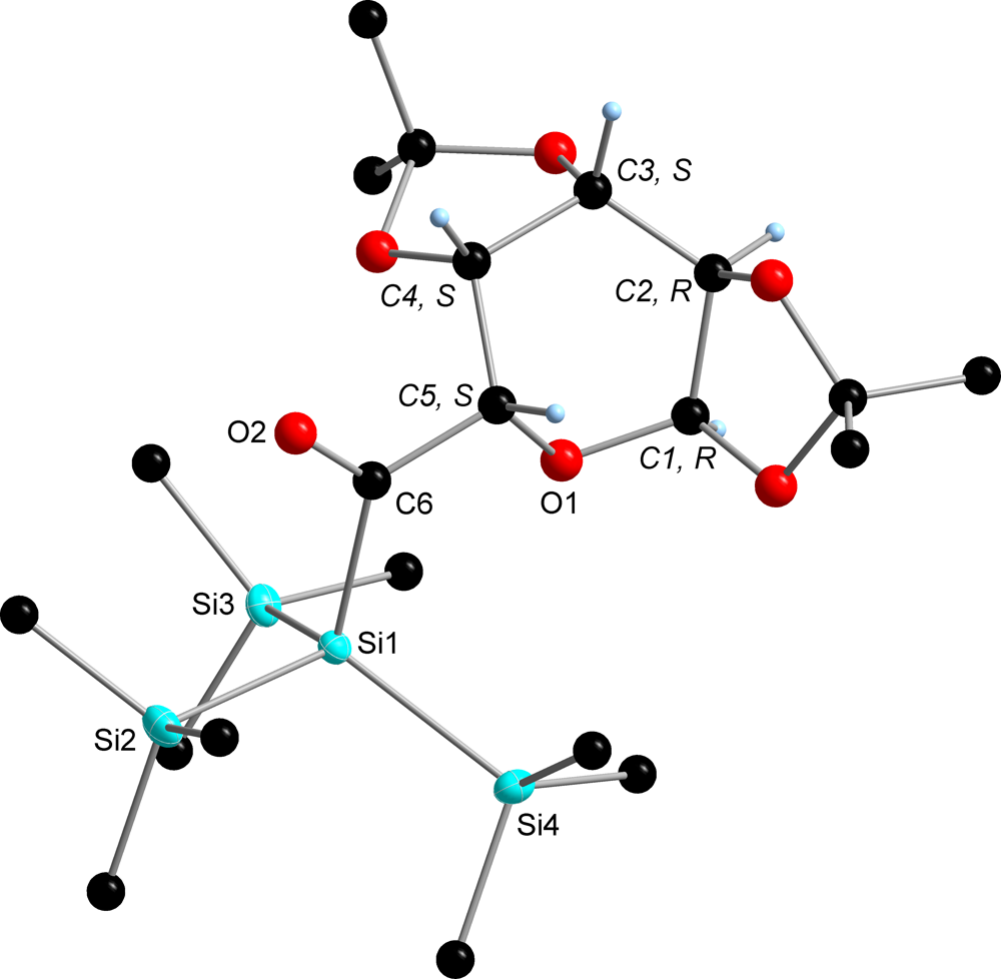
ORTEP representation for compound **1**. Thermal ellipsoids are depicted at the 50% probability level. Hydrogen atoms are omitted for clarity. Selected bond lengths (Å) with estimated standard deviations: Si(1)–Si(4) 2.3549 (14), Si(1)–Si(2) 2.3622 (14), Si(1)–Si(3) 2.3570 (14), Si(1)–C(6) 1.952 (4), and O(2)–C(6) 1.222(4).

**Figure 2:**
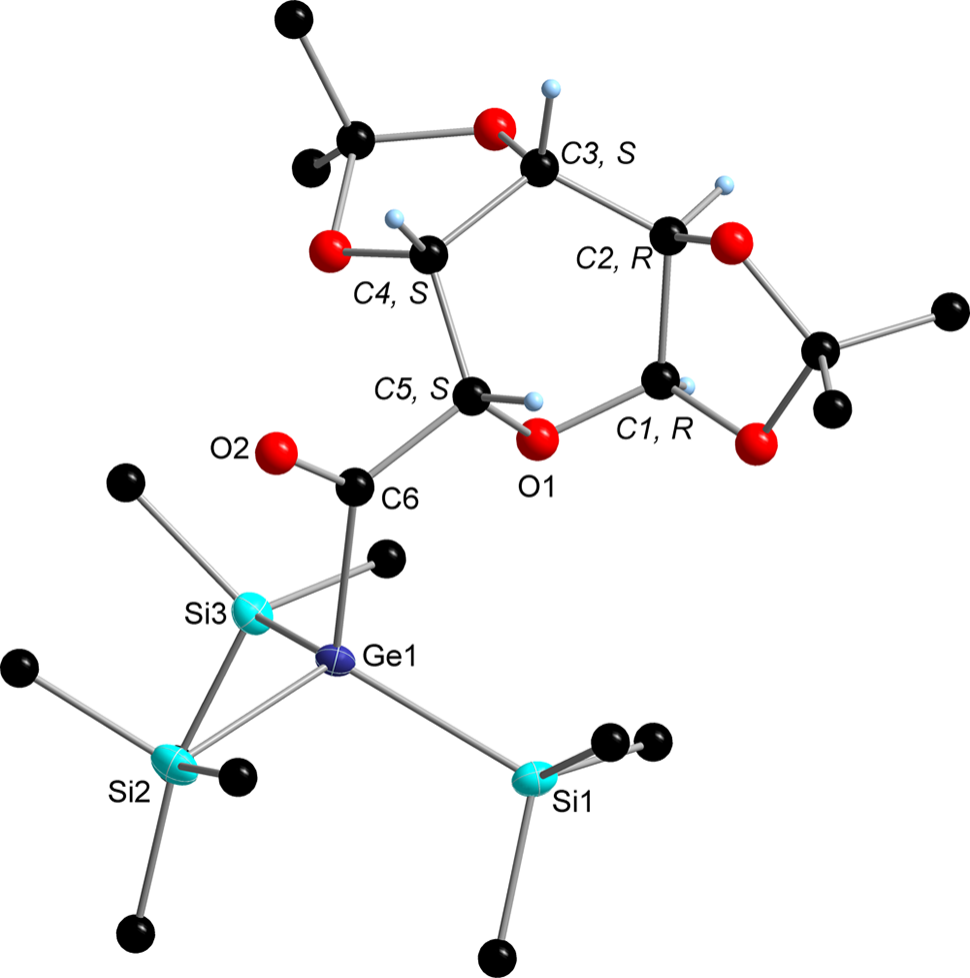
ORTEP representation for compound **2**. Thermal ellipsoids are depicted at the 50% probability level. Hydrogen atoms are omitted for clarity. Selected bond lengths (Å) with estimated standard deviations: Ge(1)–Si(1) 2.3789 (9), Ge(1)–Si(2) 2.3809 (8), Ge(1)–Si(3) 2.3822 (8), Ge(1)–C(6) 2.024 (2), and O(2)–C(6) 1.416 (3).

To determine the position of the longest wavelength absorption, the UV/vis absorption spectra of compounds **1** and **2** were recorded and compared to a structurally related monoacylgermane **3** (1,1,1-tris(trimethylsilyl)benzoylgermane).

Both compounds show long-wavelength absorptions bands with λ_max_ values between 325 and 400 nm tailing into the visible light region. Compared to compound **3**, an example of an aryl-substituted acylgermane, a significantly hypsochromic shift occurs (Figure 3). This is not surprising because it is well-known that aliphatic substituents at the carbonyl moieties undergo the same hypsochromic shift.^21,22^ Moreover, the absorption spectrum of **1** and **2** exhibits considerable fine structure consisting of four main bands which is not unusual and parallels the behavior of structurally related acyl group 14 compounds.^23^ Nevertheless, to synthesize high performance visible light photoinitiators, these blue-shift significantly, lowering the activity of these derivatives, when visible light is used.

**Figure 3:**
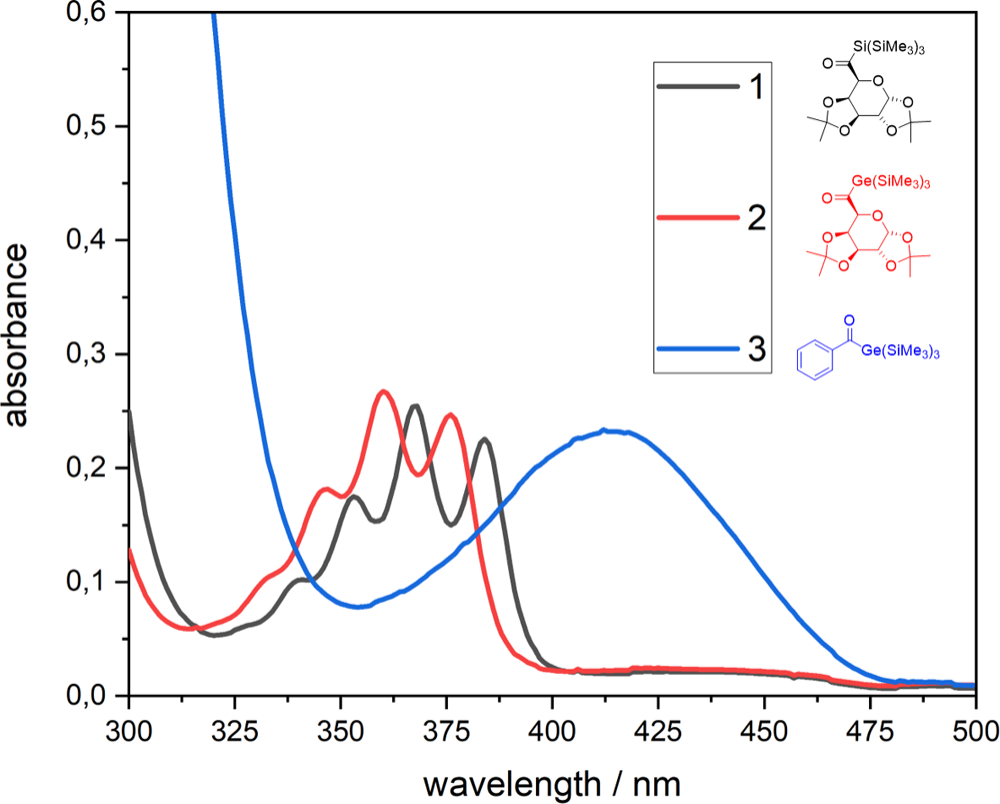
Absorption spectrum of compounds **1** and **2** compared with the monoacylgermane **3** with a concentration of 1 × 10^–3^ M in acetonitrile.

On the basis of these results, we introduced a phenyl spacer at the C_6_ position of the d-galactose moiety. The starting point of our manipulations was a Mitsunobu reaction of the 1,2:3,4-di-*O*-isopropylidene-α,d-galactose with methyl 4-hydroxybenzoate under standard Mitsunobu reaction conditions to give the respective 6-*O*-(4-methyloxycarbonylphenyl)-d-galactose moiety **4** in good yields. The analytical data are consistent with the proposed structure and can be found in the Supporting Information. The next step was the hydrolysis of the terminal methyl ester in compound **4** by saponification under conventional Zemplén conditions which led to the 6-*O*-benzoic acid d-galactose derivative **5**. The desired acid fluoride d-galactose moiety **6** was synthesized by the reaction of the carboxylic acid **5** with an equimolar amount of diethylaminosulfur trifluoride (DAST) in dichloromethane in excellent yields (Scheme 2). Again the analytical data for **5** and **6** are presented in the Supporting Information.

**Scheme 2:**
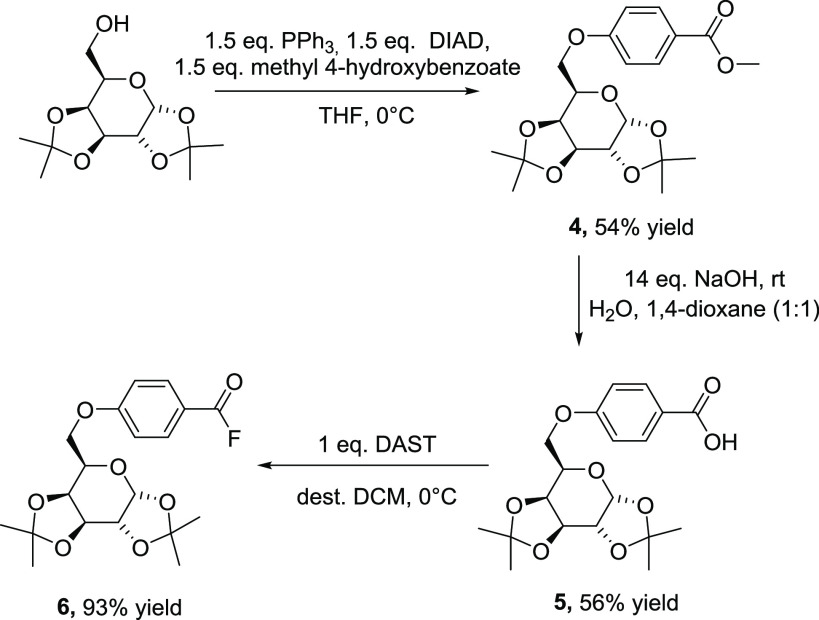
Synthetic Pathway toward **6**

The next reaction step was the attempted multiple silyl abstraction leading to tetraacylgermane **7**. Therefore, the anion KGe(SiMe_3_)_3_ (**2K**) was reacted with 4.1 mol equiv of the corresponding acid fluoride **6**. The reaction sequence used for the preparation of tetraacylgermane **7** is depicted in Scheme 3. (Note: it is not possible to synthesize tetraacylsilanes via the multiple silyl abstraction methodology. Therefore, from here on we will only focus on germanium as central atom.)

**Scheme 3:**
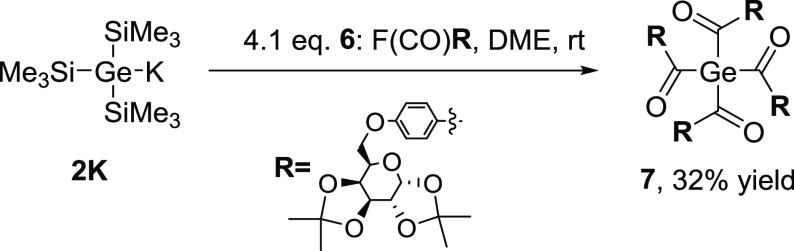
Synthetic Pathway toward **7**

The air-stable, yellow, crystalline target compound **7** was obtained in 32% yield. Analytical and spectroscopic data obtained for **7** are consistent with the proposed structure. NMR spectra and detailed assignments are provided in the Supporting Information. Compound **7** shows very similar ^13^C chemical shifts for the carbonyl C atoms at δ 219.9 ppm, which is characteristic for carbonyl groups directly linked to a germanium atom. To further elucidate substituent effects on the absorption behavior of **7**, a UV/vis absorption spectrum was recorded and compared to a structurally related tetraacylgermane **8** (tetra-*o*-toluoylgermane). We choose to compare compounds **7** with **8** because this tetraacylgermane shows “the best” absorption properties (significant bathochromically shifted absorption edge) and, more importantly, the highest solubility of all hitherto reported tetraacylgermanes. Structure **7** exhibits the longest-wavelength absorption band (n/σ–π* transition) between 350 and 450 nm [λ_max_ = 393 nm, ε ≈ 1753 mol L^–1^ cm^–1^)] tailing well into the visible region (compare Figure 4). On the one hand, the extinction coefficient of the n/σ–π* transition of compound **7** is significantly higher than in comparison to compound **8**. But on the other hand, the band is slightly hypsochromic shifted. This is well in line with the spectroscopic properties of other previously isolated and characterized tetraacylgermanes. To determine a solvatochromism, we measured the UV/vis absorption spectrum **7** in various solvents, which can be found in the Supporting Information. In line with previous observations,^20−22^ no solvatochromic effect was found.

**Figure 4:**
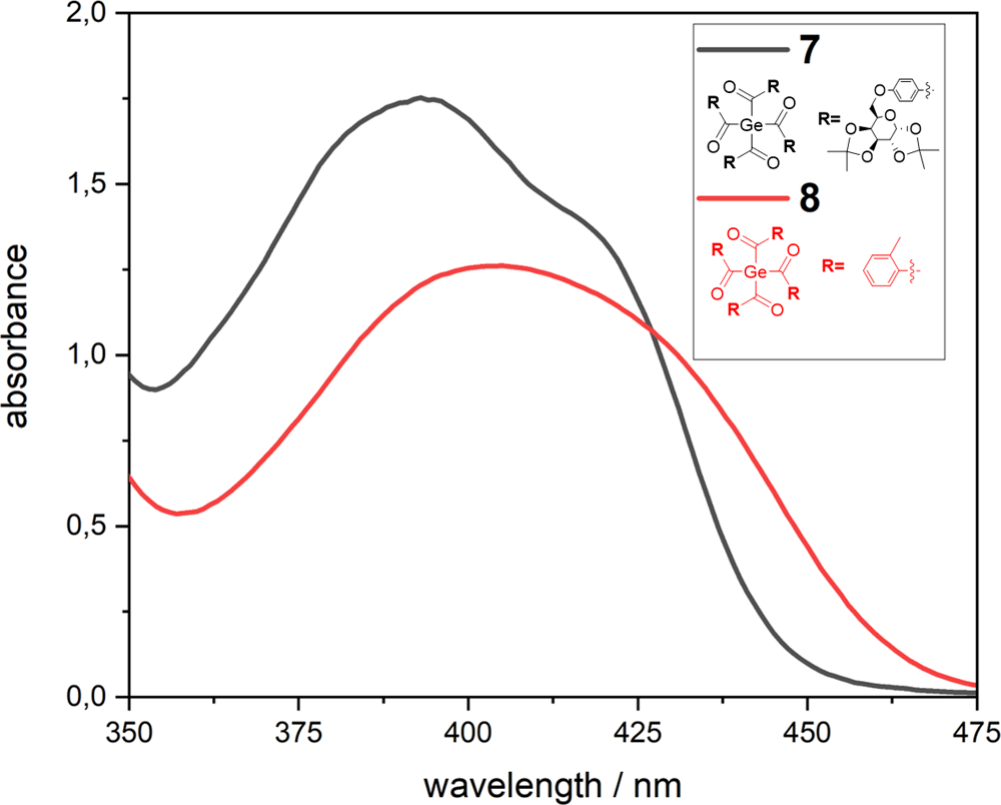
Absorption spectrum of **7** and **8** with a concentration of 1 × 10^–3^ M in acetonitrile.

The low-solubility of tetraacylgermane is a huge drawback for multiple applications. By introducing a di-*O*-isopropylidene-protected d-galactose component as a substituent, we hoped to increase the solubility in a variety of monomers. Therefore, solubility tests with methyl methacrylate (MMA), ethanol, methanol, and acetonitrile were accomplished. 100 μL of the respective solvent was used, and the amounts of dissolved compounds were analyzed. To compare the increased solubility, we again choose **8** as benchmark compound. The results obtained are summarized in Table 1. Compound **7** shows significantly higher solubilities in polar solvents. In ethanol and methanol an increase of ∼106% was observed. Moreover, in acetonitrile the solubility of **7** increases by ∼3125% compared to **8**.

**Table 1:** Amount of Compound Dissolved in 100 μL of the Respective Solvent Compared with the Tetraacylgermane **8**

	**7** [mg]	**8** [mg]	increase [%]
ethanol	1.30	0.63	106
methanol	1.00	0.56	79
acetonitrile	22.25	0.69	3125
methyl methacrylate	35.40	3.81	829

As envisaged in the introduction, deprotection of the isoproylidene groups at the sugar moiety should lead to water-soluble photoinitiators. However, despite all efforts we have not been able to isolate partly deprotected or fully deprotected acyl group 14 compounds by selective hydrolysis of the isopropylidene groups. Several standard methods under acidic conditions employing aqueous HCl, ion-exchange resin IR 120 H^+^ as well as acetic acid anhydride in various solvent mixtures did not lead to sufficient clean compounds for characterization as well as property evaluation (for experimental details see the Supporting Information). Therefore, we aim for changing the protecting group strategy on the sugar moiety to continue our studies in a follow-up report.

To conclude, we were able to successfully synthesize novel d-galactose-modified acylsilanes and acylgermanes in good to moderate yields. We first synthesized a monoacylsilane **1** and a monoacylgermane **2**. The acyl moiety bears a di-*O*-isopropylidene-protected d-galactose moiety. Both compounds show long-wavelength absorptions bands with λ_max_ values between 325 and 400 nm tailing into the visible light region. However, to synthesize high performance visible light photoinitiators, these blue-shift significantly, lowering the activity of these derivatives. Therefore, we introduced a phenyl spacer at the C_6_ position of the d-galactose moiety. Subsequently, the synthesized acid fluoride **6** was reacted with **2K**, and after multiple silyl abstractions tetraacylgermane **7** was obtained. Compound **7** exhibit the longest-wavelength absorption band between 350 and 450 nm, tailing well into the visible region. Moreover, the d-galactose-substituted tetraacylgermane **7** shows significantly higher solubility in polar solvents related to germanium-based photoinitiators, which is one step closer to the free radical polymerization in aqueous media. Synthetic approaches toward water-soluble compounds in this respect are currently in progress.

## Supporting Information Available

Experimental details, single-crystal X-ray diffraction analysis, and NMR spectra of isolated compounds (PDF)

## Accession Codes

CCDC 2041244–2041247 contain the supplementary crystallographic data for this paper. These data can be obtained free of charge via www.ccdc.cam.ac.uk/data_request/cif, or by emailing data_request@ccdc.cam.ac.uk, or by contacting The Cambridge Crystallographic Data Centre, 12 Union Road, Cambridge CB2 1EZ, UK; fax: +44 1223 336033.
