# Supplementary material for: Synthesis of d-Galactose-Substituted Acylsilanes and Acylgermanes. Model Compounds for Visible Light Photoinitiators with Intriguing High Solubility
Source: Organometallics. 2021 Apr 27;40(9):1185–9. doi: 10.1021/acs.organomet.0c00753 (PMC8155559; doi:10.1021/acs.organomet.0c00753)
Supplement: Supplementary file 1 — om0c00753_si_001.pdf [file om0c00753_si_001.pdf]

Supporting Information

Synthesis of D-Galactose Substituted Acylsilanes and Acylgermanes. Model Compounds for Visible Light Photoinitiators with Intriguing High Solubility

Lukas Schuh,† Philipp Müller, † Ana Torvisco,† Harald Stueger, Tanja M. Wrodnigg‡* and Michael Haas†*

† Institute of Inorganic Chemistry, Graz University of Technology, Stremayrgasse 9, 8010 Graz (Austria)

‡Institute of Chemistry and Technology of Biobased Systems, Graz University of Technology, Stremayrgasse 9, A-8010 Graz, Austria

Table of Content

1. Experimental Section 3
1.1 Synthesis of compound **1**..... 3
1.2 Synthesis of compound **2**..... 4
1.3 Synthesis of compound **4**..... 4
1.4 Synthesis of compound **5**..... 5
1.5 Synthesis of compound **6**..... 5
1.6 Synthesis of compound **7**..... 6
1.7 Deprotectionexperiments:..... 7
2 NMR-Spectroscopy 9
3 UV/Vis-Spectroscopy..... 16
4 Single Crystal X-ray Crystallography 17
5. References:..... 19

1. Experimental Section

All experiments were performed under a nitrogen atmosphere using standard Schlenk techniques. Solvents were dried using a column solvent purification system.[1] KO*t*Bu (>98%), 1,2;3,4-di-*O*-isopropylidene-α,D-galactose (99%), PPH_3 (for synthesis), NaOH (pellets), 1,4-dioxane (99%), diisopropyl azodicarboxylate (DIAD, 98%), IR 120 H^+ and diethylaminosulfur trifluoride (DAST, 98%) were commercially available and used without further purification. ^1H, ^{13}C and ^{29}Si NMR spectra were recorded on either a Varian INOVA 300 spectrometer and Bruker (Billerica, MA, USA) Ultrashield spectrometer at 300.36 (^1H) and 75.53 (^{13}C) MHz, respectively in C_6D_6 or $CDCl_3$ solutions and referenced versus TMS using the internal ^2H-lock signal of the solvent. HRMS spectra were performed on a Kratos Profile mass spectrometer. Infrared spectra were obtained on a Bruker Alpha-P Diamond ATR Spectrometer from the solid sample. Melting points were determined using Stuart SMP50 apparatus and are uncorrected. Elemental analyses were carried out on a Hanau Vario Elementar EL apparatus. UV absorption spectra were recorded on a Perkin Elmer Lambda 5 spectrometer

1.1 Synthesis of compound **1**

Tetrakis(trimethylsilyl)silane (1.00 g,3.10 mmol) was dissolved in 10 mL dimethoxyethane and subsequently KO*t*Bu (1M in DME, 3.42 mL,3.30 mmol) was added. After 1 hour the mixture was added dropwise to a solution of 1,2:3,4-di-*O*-isoprpylidene-α,D-galaturonic acid chloride[2] (1.00 g, 3.40 mmol) in 50 mL DME at -70 °C. During the addition, the reaction mixture went from colorless to yellow. The reaction was allowed to come to room temperature and was stirred overnight, a color change from yellow to colorless occurred. The mixture was neutralized with a satd. $NaHCO_3$ solution and the organic layer was separated and dried over Na_2SO_4. After removing the solvents under reduced pressure the product was purified by silica gel chromatography (cyclohexane:ethylacetate; 3:1). Yield: 1.20 g (75%) of analytically pure **1** as colorless powder.

1: mp: 87-88 °C. **Anal. Calc.** for $C_{21}H_{44}O_6Si_4$ C 49.95; H 8.78% Found: C 50.25; H 8.48%.
^{1}H-NMR (C_6D_6, TMS, ppm) 5.41 (d, 1H, $J_{1,2}$ = 4.9 Hz, H-1gal), 4.52 (dd, 1H, $J_{3,4}$ = 8.1 Hz, $J_{2,3}$ = 1.8 Hz, H-3gal), 4.26 (dd, 1H, $J_{4,5}$ = 1.8 Hz, H-4gal), 4.01 (dd, 1H, H-2gal), 3.87 (d, 1H, H-5gal), 1.36; 1.23; 0.93; 0.91 (s, 12H, 2xC(C$\underline{H}_3$)$_2$), 0.4 (s, 27H, Si(CH$_3$)$_3$). **^{13}C-NMR** (C_6D_6, TMS, ppm) 245.97 (**C**=O) 108.32; 107.81 (2x$\underline{C}$(CH$_3$)$_2$), 96.30 (C1), 77.92; 71.69; 71.32; 70.35 (C2, C3, C4, C5), 25.76;

25.60; 24.27; 22.67 (4C, 2xC(C̲H$_3$)), 1.43 (Si(CH$_3$)$_3$). **^{29}Si-NMR** (C_6D_6, TMS, ppm) -11.20 (Si(CH$_3$)$_3$), -70.06 (Si, q). **HRMS:** for [$C_{21}H_{44}O_6Si_4$ – CH_3] calc. 489.1980 found: 489.1984. **IR:** ν [cm-1] = 2943, 1630 (C=O), 1384, 1239, 1063, 1000, 827, 683, 623. **UV−vis:** λ [nm] (ε [L mol−1 cm−1]): 264 (2549), 344 (172), 358 (255), 374 (237).

1.2 Synthesis of compound **2**

Tetrakis(trimethylsilyl)germane (1.51 g, 4.10 mmol) was dissolved in 10 mL dimethoxyethane. and KO*t*Bu (1M in DME, 4.13 mL, 4.30 mmol) was added. After 1 hour the mixture was added dropwise to a solution of 1,2:3,4-di-*O*-isoprpylidene-α,D-galaturonic acid chloride[2] (1.33 g, 4.50 mmol) in 50 mL DME at -70 °C. During the addition, the reaction mixture went from colorless to yellow. The reaction was allowed to come to room temperature and was stirred overnight, a color change from yellow to colorless occurred. The mixture was neutralized with a satd $NaHCO_3$ solution and the organic layer was separated and dried over Na_2SO_4. The solvents were removed under reduced pressure and the product was purified by silica gel chromatography (cyclohexane:ethylacetate; 3:1). Yield: 0.90 g (40%) of analytically pure **2** as colorless powder.

2: mp: 88-89 °C. **Anal. Calc.** for $C_{21}H_{44}O_6Si_3Ge$ C 45.91; H 8.07% Found: C 45.84; H 7.81%.
^{1}H-NMR (C_6D_6, TMS, ppm) 5.42 (d, 1H, $J_{1,2}$ = 4.9 Hz, H-1gal), 4.53 (dd, 1H, $J_{2,3}$ = 1.8 Hz, $J_{3,4}$ = 8.1 Hz, H-3gal), 4.26 (dd, 1H, $J_{4,5}$ = 1,8 Hz, H-4gal), 4.01 (dd, 1H, H-2gal), 3.82 (d, 1H, H-5gal), 1.36; 1.23; 0.94; 0.92 (s, 12H, 2xC(CH̲$_3$)$_2$), 0.42 (s, 27H, Si(CH$_3$)$_3$). **^{13}C-NMR** (C_6D_6, TMS, ppm) 242.52 (C=O) 105.86; 105.31 (2xC̲(CH$_3$)$_2$), 93.91 (C1), 75.28; 69.24; 68.88; 67.98 (C2, C3, C4, C5), 23.29; 23.16; 21.80; 20.25 (4C, 2xC(C̲H$_3$)$_2$), 1.43 (Si(CH$_3$)$_3$). **^{29}Si-NMR** (C_6D_6, TMS, ppm) -4.85 (Si(CH$_3$)$_3$. **HRMS:** for $C_{21}H_{44}O_6Si_3Ge$ calc. 550.1662 found: 550.1654. **IR:** ν [cm-1] = 2943, 1643 (C=O), 1384, 1232, 1211, 1070, 1000, 824, 690, 627. **UV−vis:** λ [nm] (ε [L mol−1 cm−1]): 257 (2330), 351 (164), 367 (244), 383 (222).

1.3 Synthesis of compound **4**

To a solution of 1,2:3,4-di-*O*-isopropylidene-α,D-galactopyranose (13.00 g, 49.90 mmol) in THF (200 mL) PPh_3 (19.65 g, 74.90 mmol) was added at 0 °C. Subsequently, diisopropyl azodicarboxylate (DIAD) (15.15 g, 74.90 mmol) was added and the solution turned yellow. After 5 minutes a colorless solid precipitated. After the addition of methoxy-4-hydroxibenzoate (11.40 g, 74.90 mmol), the solid re-dissolves in the solution and turned yellowish. The reaction was allowed to come to room temperature which was stirred for 3 days. The mixture was neutralized by the addition of a satd $NaHCO_3$ solution, the organic layer was separated and dried over Na_2SO_4. After evaporation of the

solvents the product was purified by silica gel chromatography (cyclohexane:ethylacetate; 20:1). Yield: 10.61 g (54%) of analytically pure **4** as colorless crystals.

4: mp: 87-88 °C. **Anal. Calc.** for $C_{20}H_{26}O_8$ C 60.90; H 6.64% Found: C 60.49; H6.47%.
^{1}H-NMR ($CDCl_3$, TMS, ppm) 7.97 (d, 2H, $J_{1,3}$ = 9 Hz, arom.), 6.96 (d, 2H, $J_{1,3}$ = 9 Hz, arom.), 5.56 (d, 1H, $J_{1,2}$ = 4.9 Hz, H-1gal), 4.65 (dd, 1H, $J_{2,3}$ = 2,2 Hz, $J_{3,4}$ = 7,9 Hz, H-3gal), 4.36 - 4.34 (bdd, 2H, H-4gal, H-6gal), 4.19 - 4.17 (m, 3H, H-2gal, H-5gal, H-6gal), 3.87 (s, 3H, OCH_3), 1.52; 1.46 (s, 6H, $C(C\underline{H}_3)_2$), 1.35; 1.34 (d, 6H, $C(C\underline{H}_3)_2$). **^{13}C-NMR** ($CDCl_3$, TMS, ppm) 166.96 (C=O), 162.51; 122.95 (ipso-C), 131.64; 114.47 (arom.), 109.68; 108.93 ($2x\underline{C}(CH_3)_2$), 96.49 (C1), 71.07; 70.77; 70.70 (C2, C3, C4), 66.94 (C6), 66.31 (C5), 51.96 (s, 3H, OCH_3), 26.18; 26.11; 25.05; 24.57 (4C, $2xC(\underline{C}H_3)_2$). **HRMS:** for $C_{20}H_{26}O_8$ calc. 394.1628 found: 394.1624.

1.4 Synthesis of compound **5**

Compound **4** (3.64 g, 8.90 mmol) was dissolved in a mixture of 1,4-dioxane/dest water (200 mL, v/v 1:1) and NaOH sd (4.50 g, 107.00 mmol) was added . The reaction mixture was stirred for 24 h at room temperature und the reaction progress was controlled *via* thin layer chromatography (cyclohexane/ethylacetate v/v 3:1). The mixture was neutralized by addition of $KHSO_4$ solution (5 M in water) and a soluion of satd $NaHCO_3$, the organic layer was separated, dried over Na_2SO_4 and the solvent removed under reduced pressure. The product was purified by silica gel chromatography (cyclohexane:ethylacetate; 3:1). Yield: 1.96 g (56%) of analytically pure **5** as colorless crystals.

5: mp: 95-98 °C.
^{1}H-NMR ($CDCl_3$, TMS, ppm) 11.40 (bs, 1H, COO$\underline{H}$), 7.96 (d, 2H, $J_{1,3}$ = 9 Hz, arom.), 6.89 (d, 2H, $J_{1,3}$ = 9 Hz, arom.), 5.53 (d, 1H, $J_{1,2}$ = 4.9 Hz, H-1gal), 4.62 (dd, 1H, $J_{2,3}$ = 2.3 Hz, $J_{3,4}$ = 8.8 Hz, H-3gal), 4.34 - 4.30 (m, 2H, H-4gal, H-6gal), 4.18 - 4.14 (m, 3H, H-2gal, H-5gal, H-6gal), 1.48; 1.43 (s, 6H, $C(C\underline{H}_3)_2$), 1.32; 1.30 (d, 6H, $C(C\underline{H}_3)_2$). **^{13}C-NMR** ($CDCl_3$, TMS, ppm) 171.49 (C=O), 162.46; 123.33 (ipso-C), 132.07; 114.25 (arom.), 109.52; 108.79 ($2x\underline{C}(CH_3)_2$), 96.33 (C1), 70.91; 70.61; 70.55 (C2, C3, C4), 67.03 (C6), 66.15 (C5), 26.05; 25.99; 24.93; 24.45 (4C, $C(\underline{C}H_3)_2$).

1.5 Synthesis of compound **6**

Compound **5** (1.61 g, 4.20 mmol) was dissolved in 100 mL dichlormethane at 0 °C. Diethylaminosulfur trifluoride (DAST) (1.05 ml, 6.50 mmol) was added dropwise and the solution turned yellow. After 2 h the mixture was extracted with a 0.10 M HCl. The organic layer was

separated and dried over Na_2SO_4. After evaporation of the solvents a highly viscous brown oil was obtained, which crystallized overnight. Yield: 1.55 g (93%) of analytical pure brownish crystals **6**.

6: **^{1}H-NMR** (C_6D_6, TMS, ppm) 7.68 (d, 2H, $J_{1,3}$ = 9 Hz, arom.), 6.54 (d, 2H, $J_{1,3}$ = 9 Hz, arom.), 5.46 (d, 1H, $J_{1,2}$ = 5.0 Hz, H-1gal), 4.49 (d, 1H, $J_{2,3}$ = 2,4 Hz, $J_{3,4}$ = 7.9 Hz, H-3gal), 4.20 - 4.11 (m, 2H, H-4gal, H-6gal), 4.10 – 4.00 (m, 3H, H-2gal, H-5gal, H-6gal), 1.43; 1.40 (s, 6H, 2xC(C$\underline{H}_3$)$_2$), 1.16; 1.08 (d, 6H, 2xC(C$\underline{H}_3$)$_2$). **^{13}C-NMR** (C_6D_6, TMS, ppm) 159.45; 154.95 (C=O) 164.45 (ipso-C), 133.77; 133.72 (arom.), 117.74; 116.91 (ipso-C), 115.19 (arom.), 109.50; 108.68 (2x $\underline{C}$(CH$_3$)$_2$), 96.77 (C1), 71.25; 71.14; 70.90 (C2, C3, C4), 67.69 (C6), 66.56 (C5), 26.21; 26.18; 24.84; 24.38 (4C, 2xC($\underline{C}$H$_3$)$_2$). **HRMS:** for $C_{19}H_{23}FO_7$ calc. 394.1628 found: 394.1624

1.6 Synthesis of compound **7**

Tetrakis(trimethylsilylgermane) (0.50 g, 1,37 mmol) was dissolved in 20 mL dimethoxyethane (DME). Subsequently, KO*t*Bu (1M in DME, 1.43 mL, 1,43 mmol) was added. After 1 hour the mixture was added dropwise to a solution of compound **6** (2.15 g, 5,62 mmol) in 50 mL DME at -70 °C. During the addition, the reaction mixture went from colorless to yellow. The reaction was allowed to come to room temperature and was stirred overnight. The mixture was neutralized with a $NaHCO_3$ solution and the organic layer was separated and dried over Na_2SO_4. After evaporation of the solvents the product was crystalized at -30 degrees in methanol. Yield: 0.68 mg (32%) of analytically pure **7** as yellow powder.

7: **mp:** 119-121 °C. **Anal. Calc.** for $C_{76}H_{92}O_{28}Ge$ C 59.81; H 6.08% Found: C 59.62; H 5.96%.
^{1}H-NMR (C_6D_6, TMS, ppm) 8.08 (d, 8H, $J_{1,3}$ = 9 Hz, arom.), 6.50 (d, 8H, $J_{1,3}$ = 9 Hz, arom.), 5.48 (d, 4H, $J_{1,2}$ = 6 Hz, 4xH-1gal), 4.47 (d, 4H, $J_{2,3}$ = 2,3 Hz, $J_{3,4}$ = 8.0 Hz, 4xH-3gal), 4.17 – 4.14 (m, 8H, 4xH-4gal, 4xH-6gal), 4.02 – 3.92 (m, 12H, 4xH-2gal, 4xH-5gal, 4xH-6gal), 1.45; 1.39 (s, 24H, 4xC(C$\underline{H}_3$)$_2$), 1.13; 1.04 (s, 24H, 4xC(C$\underline{H}_3$)$_2$) **^{13}C-NMR** (C_6D_6, TMS, ppm) 219.86 (C=O) 163.23; 134.74 (ipso-C), 131.70; 114.78 (arom.), 108.96; 108.19 (2x$\underline{C}$(CH$_3$)$_2$), 96.37 (C1), 70.81; 70.73; 70.56 (C3, C4, C2), 66.93 (C6), 65.99 (C5), 25.81; 25.76; 24.45; 23.99 (4C, 2xC($\underline{C}$H$_3$)$_2$). **HRMS:** for $C_{76}H_{92}O_{28}Ge$ calc. 1526.4987 found: 1526.4995 **IR:** ν [cm-1] = 2983, 2933, 1623 (C=O), 1592,1571, 1210, 1158, 1066, 999, 889, 831, 648, 507 **UV−vis:** λ [nm] (ε [L mol−1 cm−1]): 302 (208500), 397 (7020).

1.7 Deprotection Experiments:

Deprotection with hydrochlorid acid:

The respective compounds were dissolved in 10 mL dichloromethane (DCM). Subsequently, 5ml 0,1 M hydrochloric acid was added at room temperature. The reaction was controlled via NMR-spectroscopy as well as thin layer chromatography (solvent: 1:1 cyclohexane:ethyl acetate). After complete consumption of the starting material, the reaction solution was subjected to an aqueous work up. The organic layer was separated and dried over Na_2SO_4. Compound **1** (0.50 g, 0,99 mmol), compound **2** (0.50 g, 0,90 mmol), ompound **7** (0.50 g, 0,31 mmol) were reacted accordingly. According to NMR-spectroscopy no selective deprotection was observed. In all cases the only isolable compound was the respective carboxylic acid.

Deprotection with ion exchange resin IR 120 H^+:

The respective compounds were dissolved in 10 mL in a mixture of dist. water and acetonitrile (v/v 1:1) and catalytic amounts of IR 120 H^+ (washed three times with dest. H_2O) were added. Subsequently, the reactions mixture was heated to 40°C for three days. The reaction was monitored via NMR-spectroscopy as well as thin layer chromatography (solvent: 1:1 cyclohexane:ethyl acetate). Compound **1** (0.50 g, 0,99 mmol) and compound **2** (0.50 g, 0,90 mmol) were reacted accordingly. In both cases no complete consumption of the starting material was observed after three days. Prolonged stirring at this temperature and subsequent addition IR 120 H^+ led to an uncharacterizable product mixture.

Deprotection with acetic anhydride:

The respective compounds were dissolved in 10 mL acetonitrile. Subsequently, 3 ml conc. acetic anhydride was added at room temperature. Compound **1** (0.50 g, 0,99 mmol) and compound **2** (0.50 g, 0,90 mmol) were reacted accordingly. After 5 days in both cases no conversion of the starting material was observed by thin layer chromatography (solvent: 1:1 cyclohexane:ethyl acetate).

2 NMR-Spectroscopy

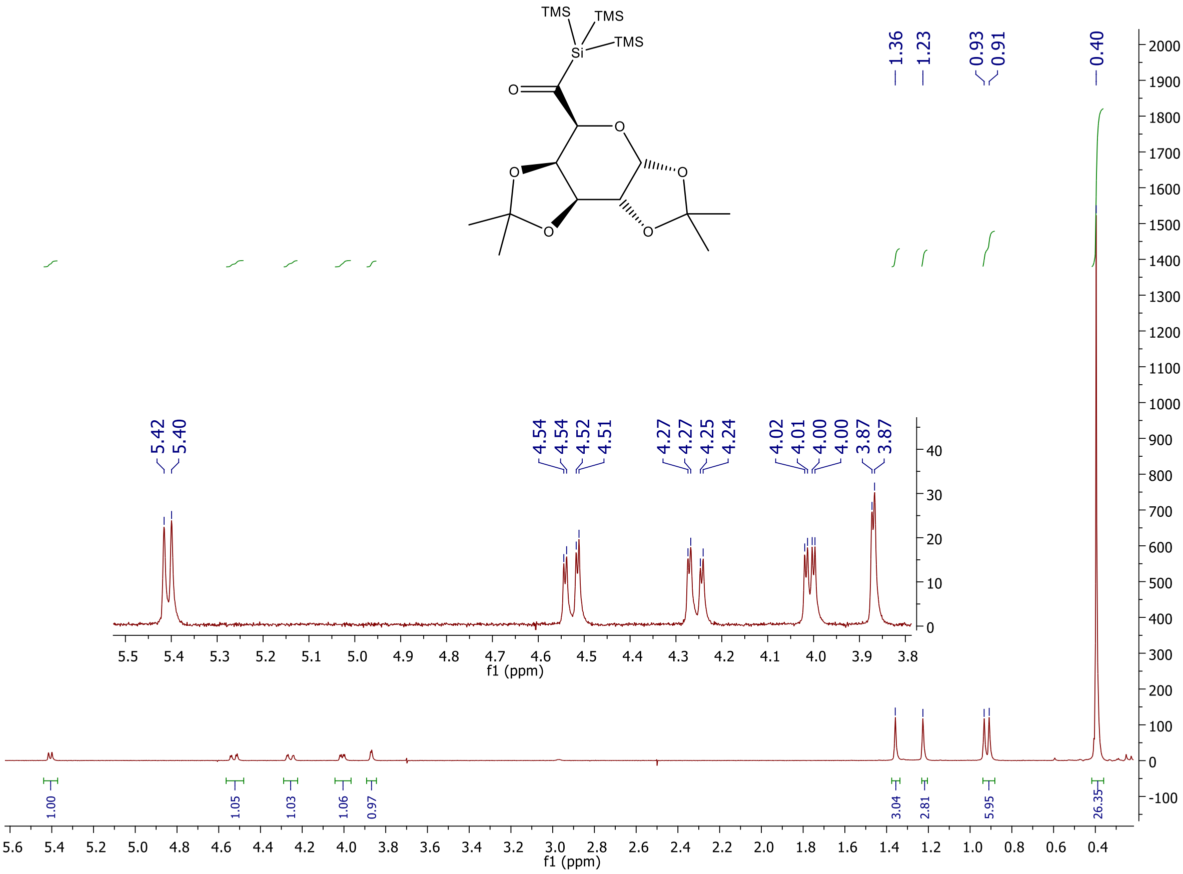

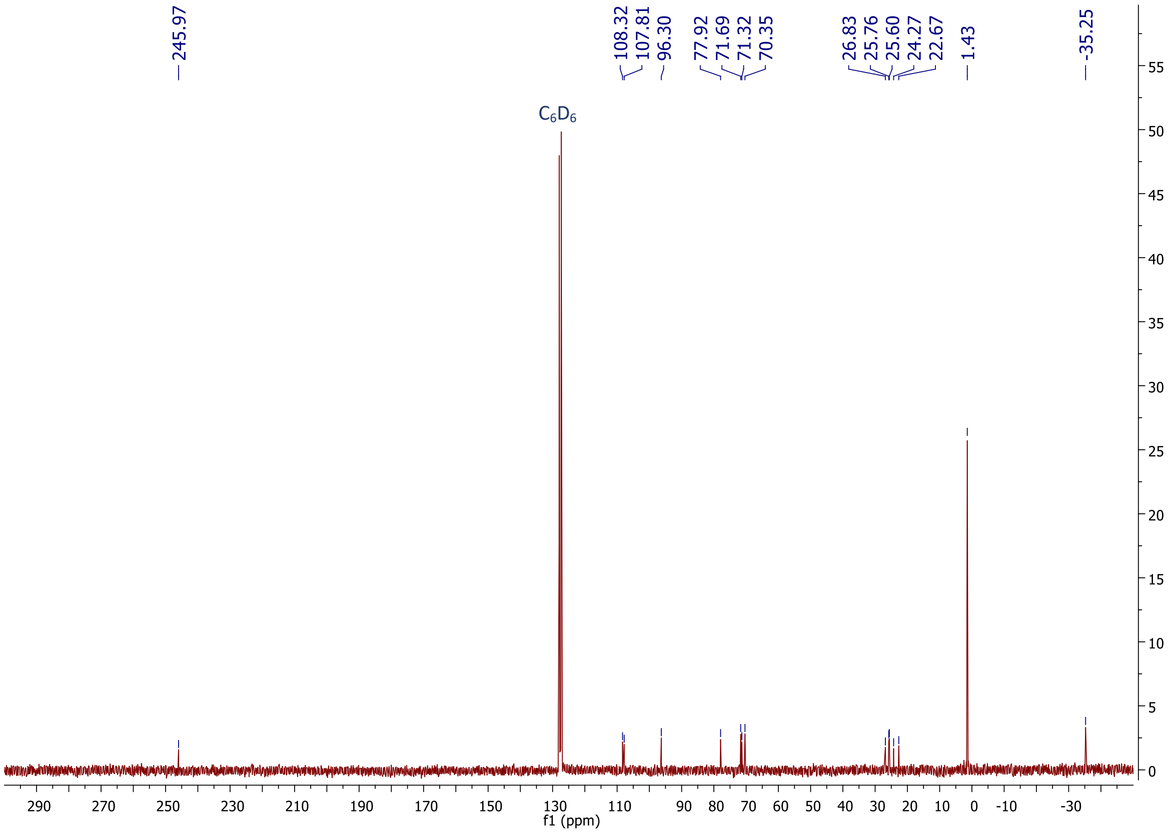

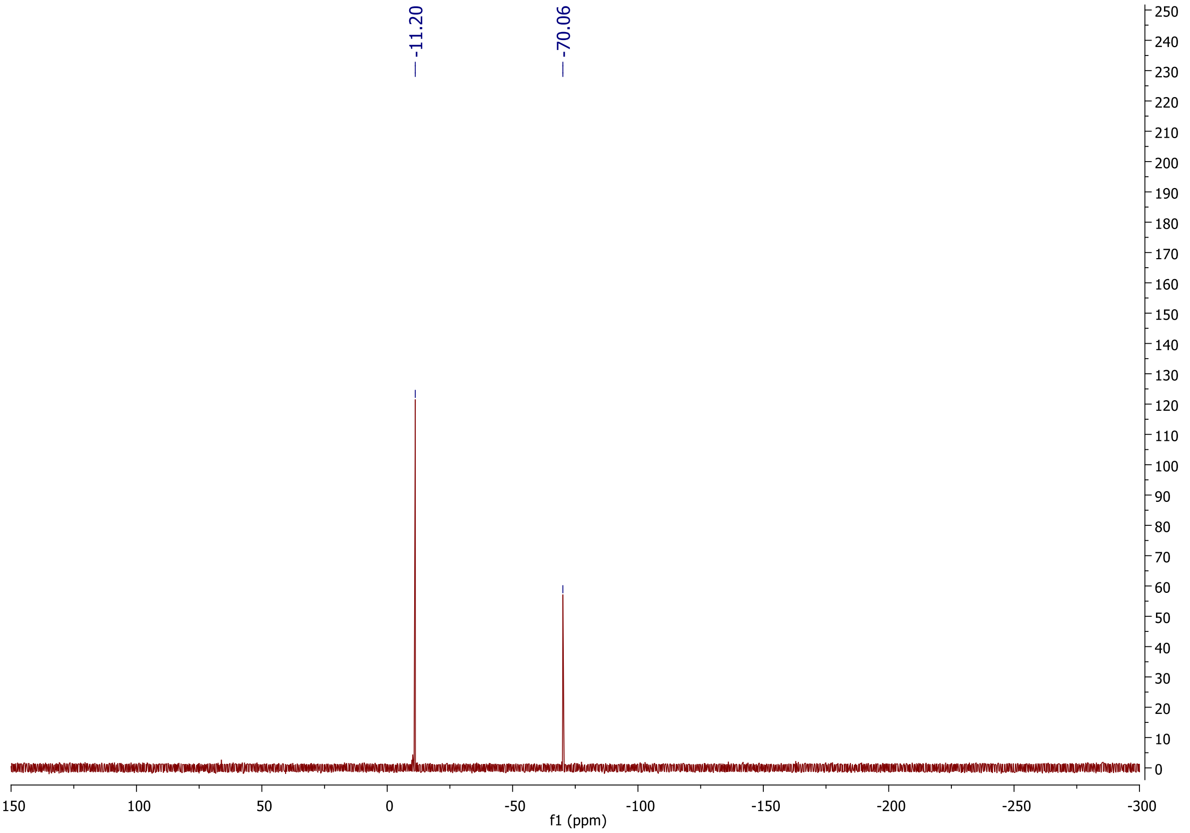

Figure 1: ^{1}H-NMR spectra, ^{13}C-NMR spectra and ^{29}Si-Inept spectra of ***1***

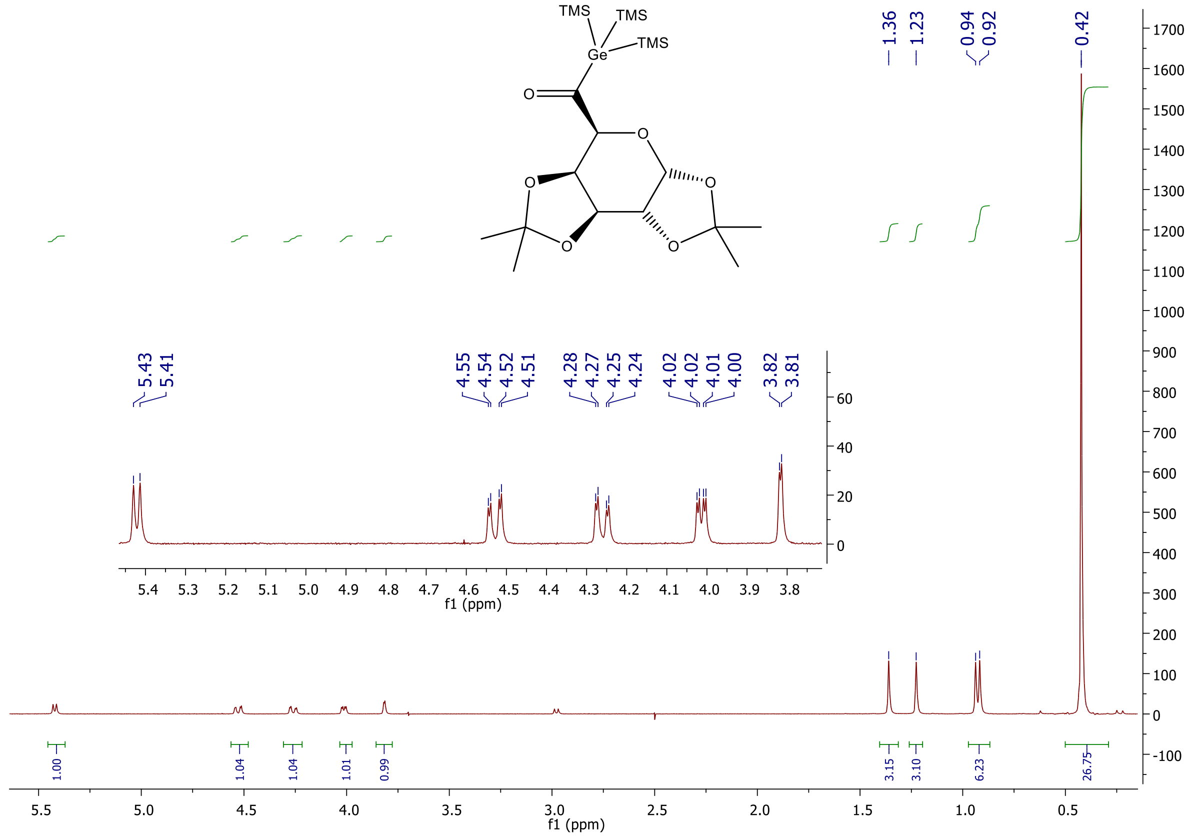

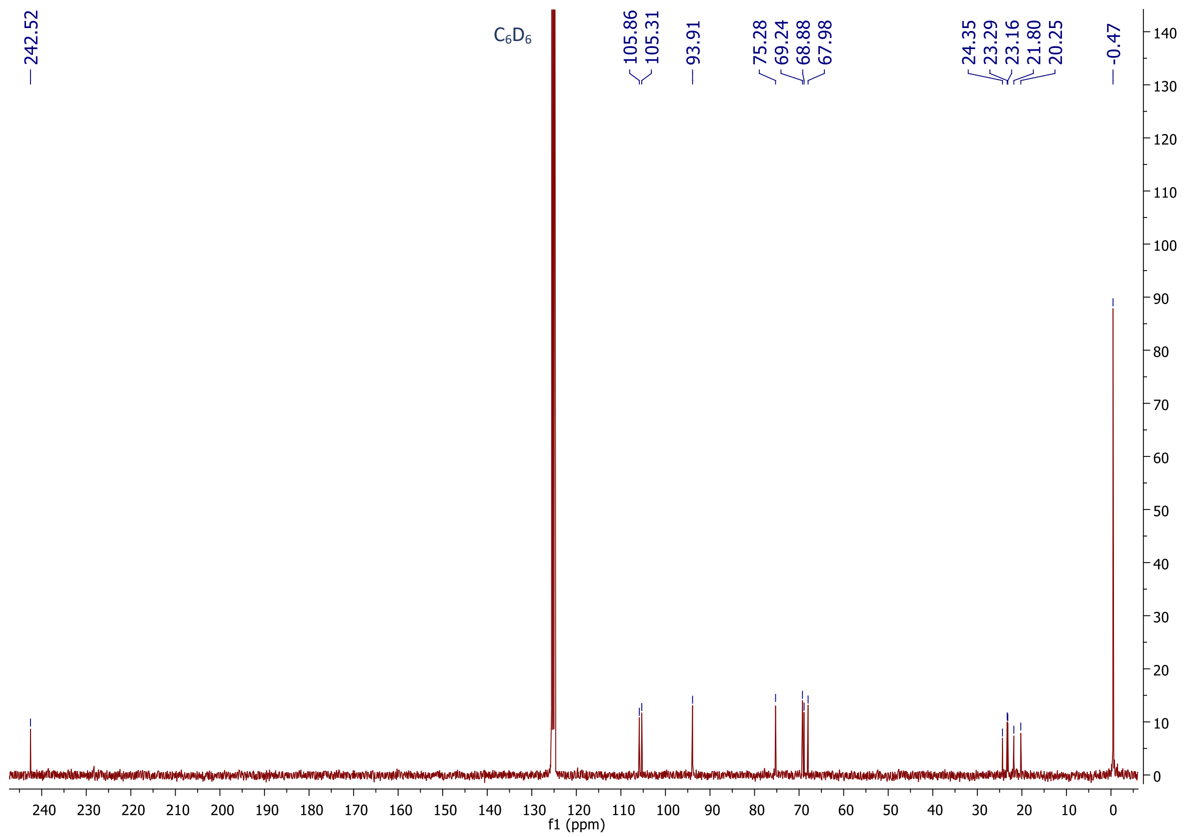

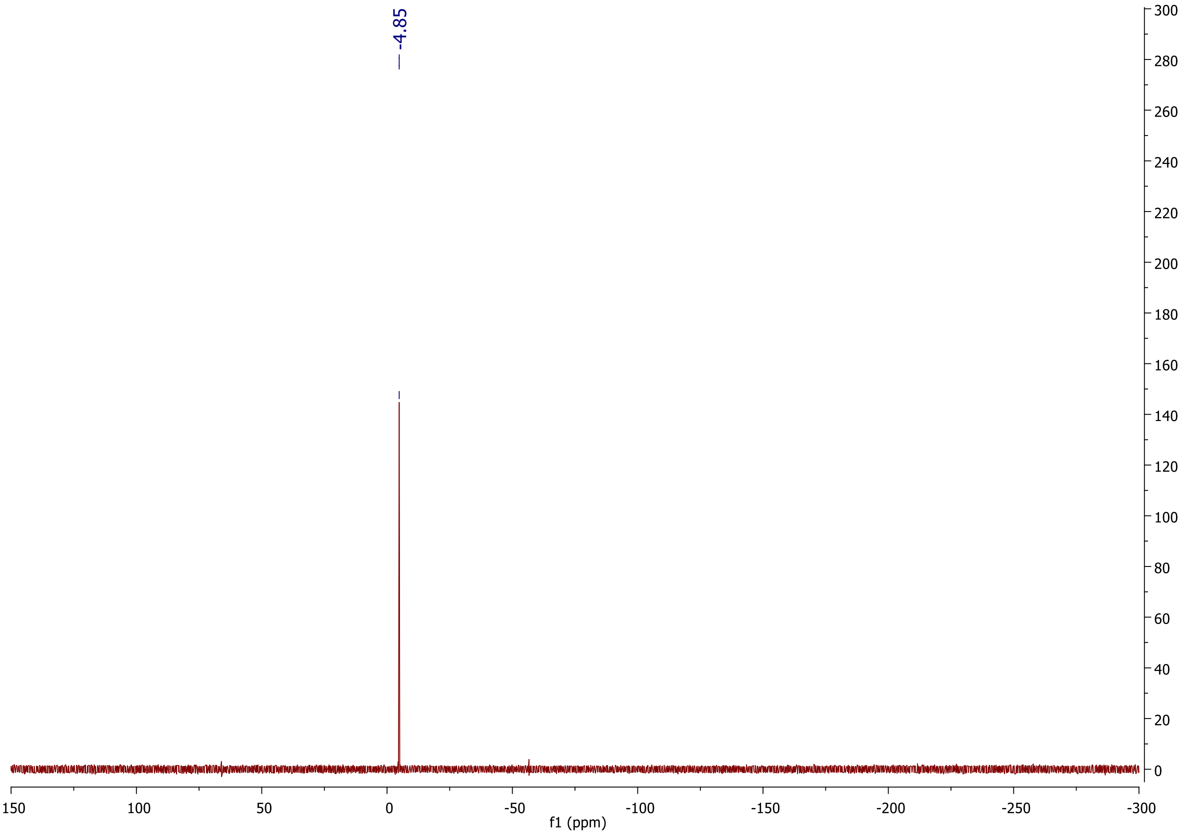

*FIgure 2: ^{1}H-NMR spectra, ^{13}C-NMR spectra and ^{29}Si-Inept spectra of **2***

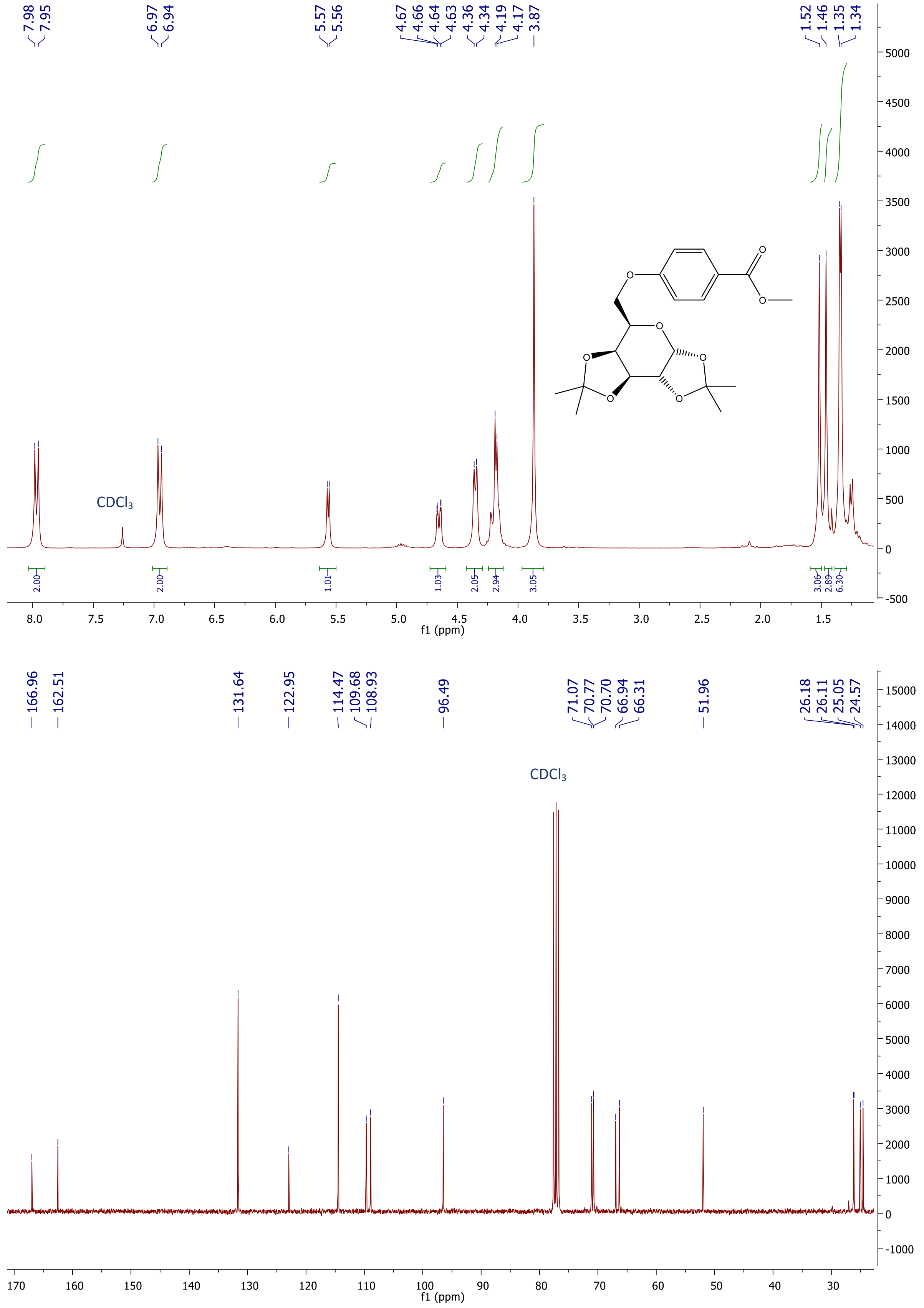

*Figure 3: ^{1}H-NMR spectra, ^{13}C-NMR spectra of **4***

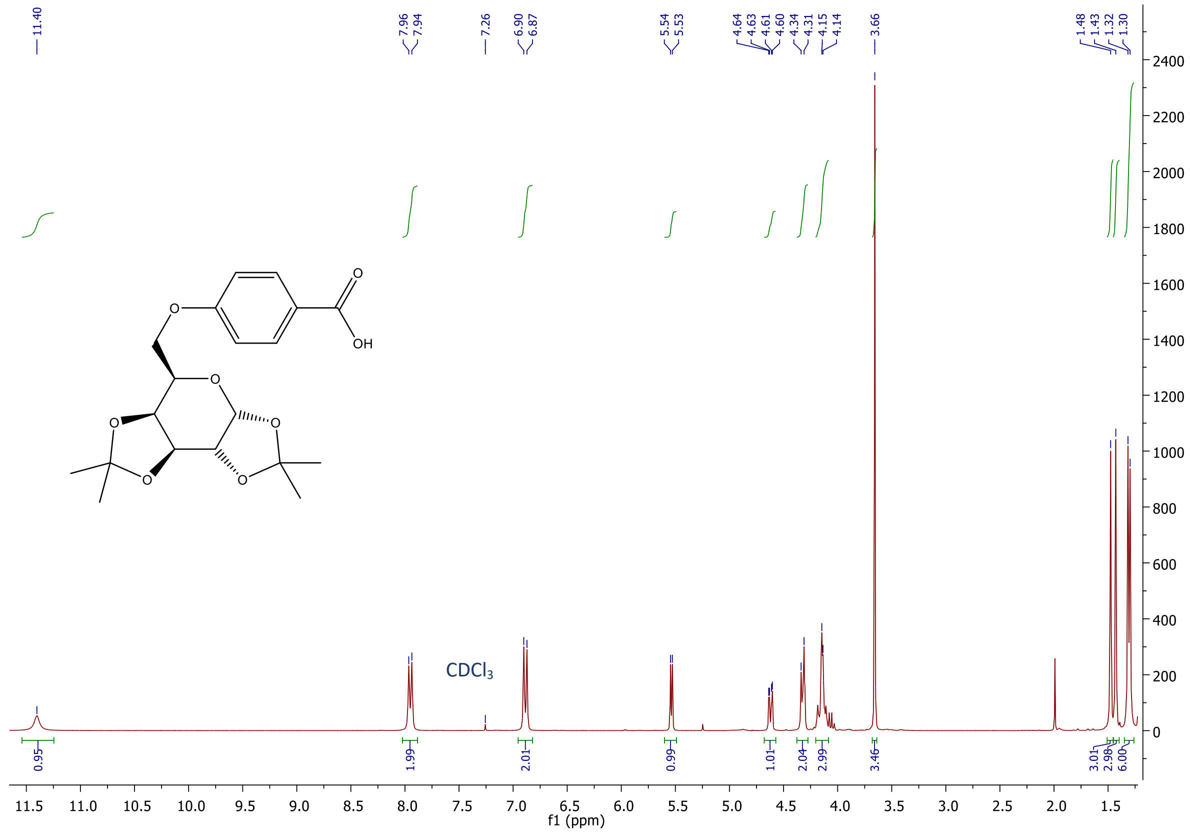

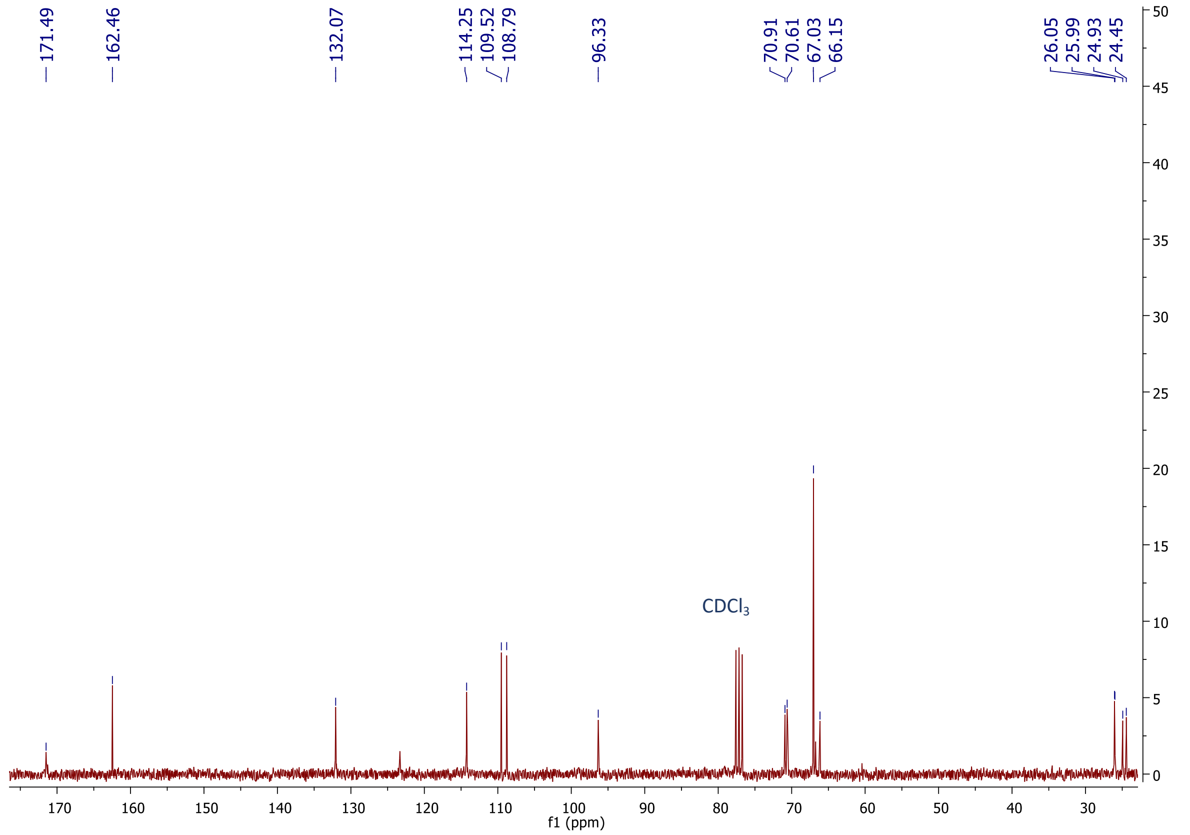

*FIgure 4: ^{1}H-NMR spectra, ^{13}C-NMR spectra of **5***

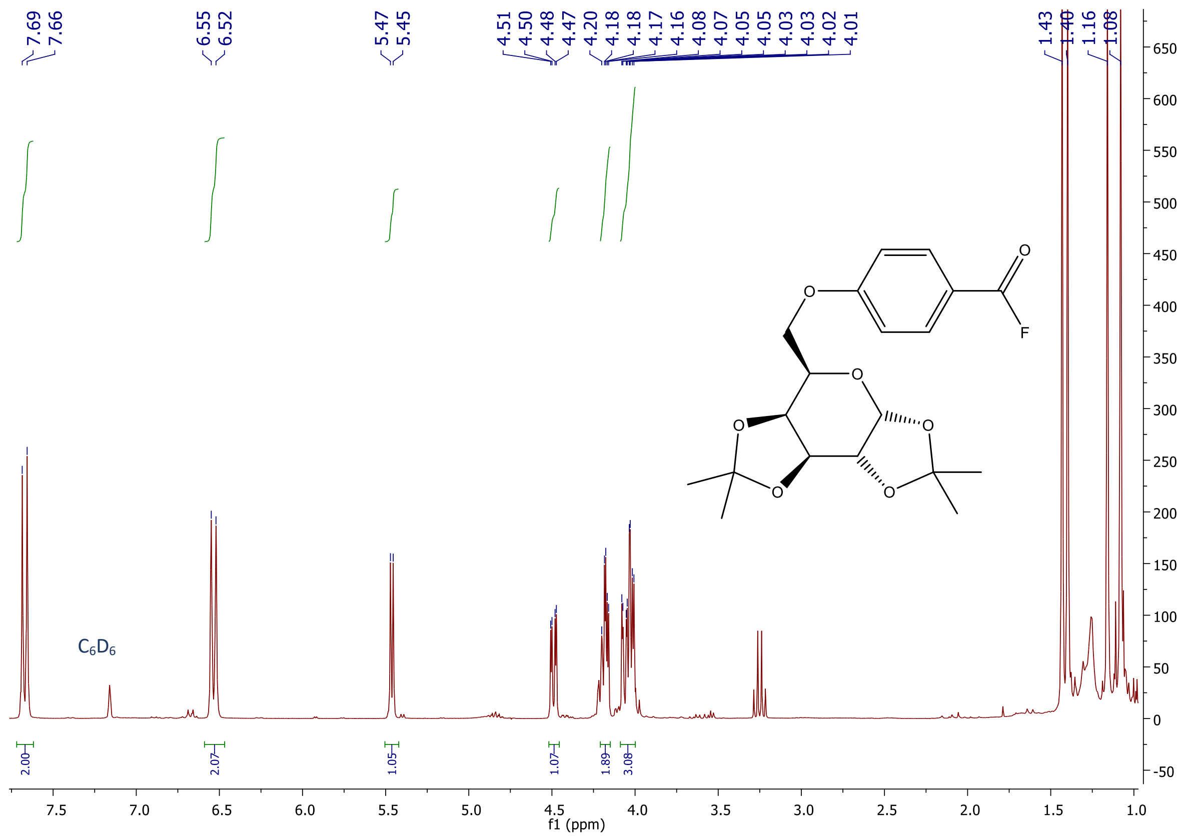
7.69 7.66 6.55 6.52 5.47 5.45 4.51 4.50 4.48 4.47 4.20 4.18 4.18 4.17 4.16 4.08 4.07 4.05 4.05 4.03 4.03 4.02 4.01 1.43 1.40 1.16 1.08
C6D6
2.00 2.07 1.05 1.07 1.89 3.08
O
F
f1 (ppm)

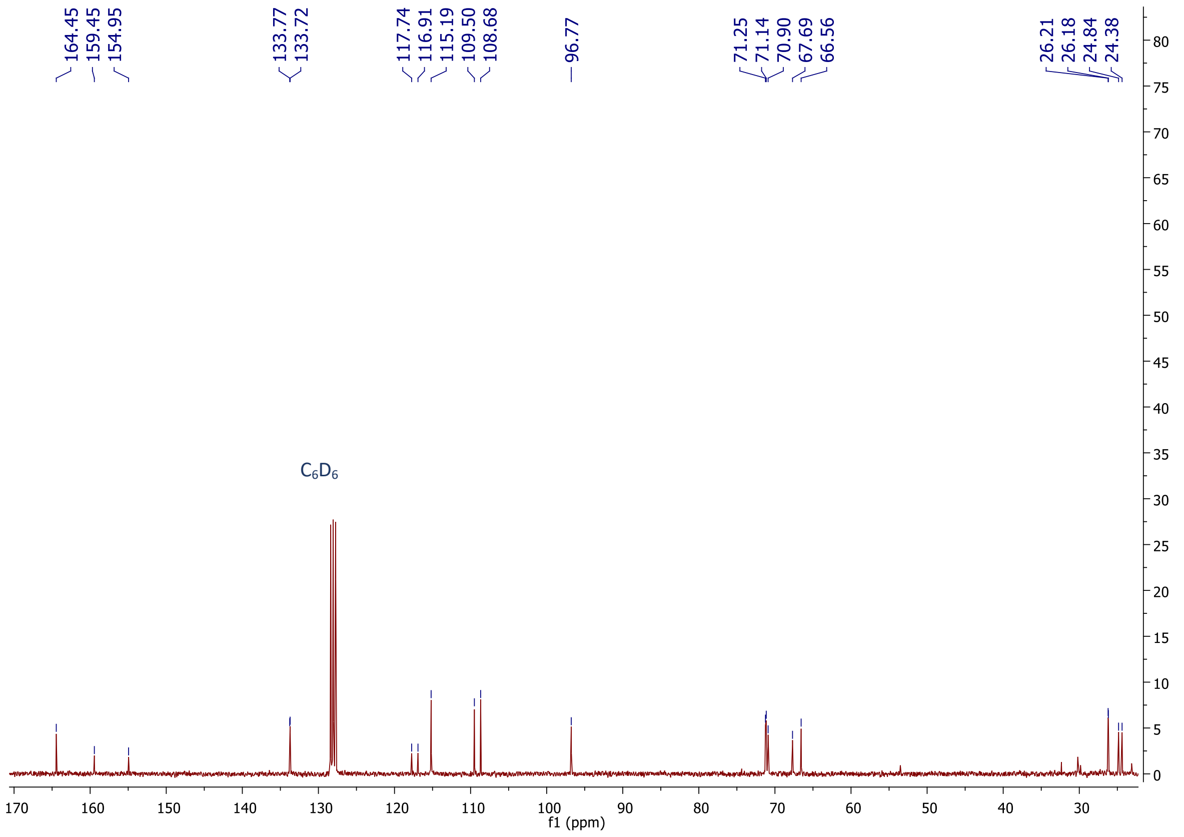
164.45 159.45 154.95 133.77 133.72 117.74 116.91 115.19 109.50 108.68 96.77 71.25 71.14 70.90 67.69 66.56 26.21 26.18 24.84 24.38
C6D6
f1 (ppm)

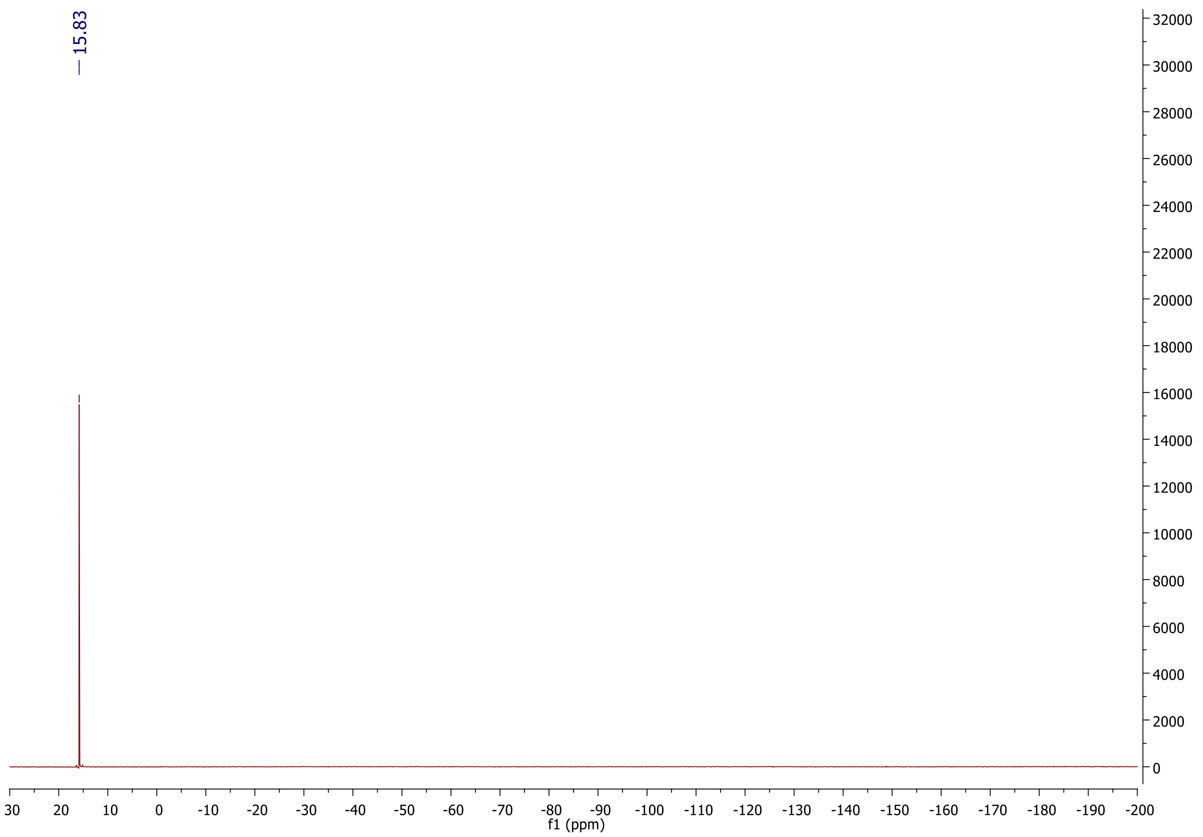

*FIgure 5: ^{1}H-NMR spectra, ^{13}C-NMR spectra and ^{19}F-NMR spectra of **6***

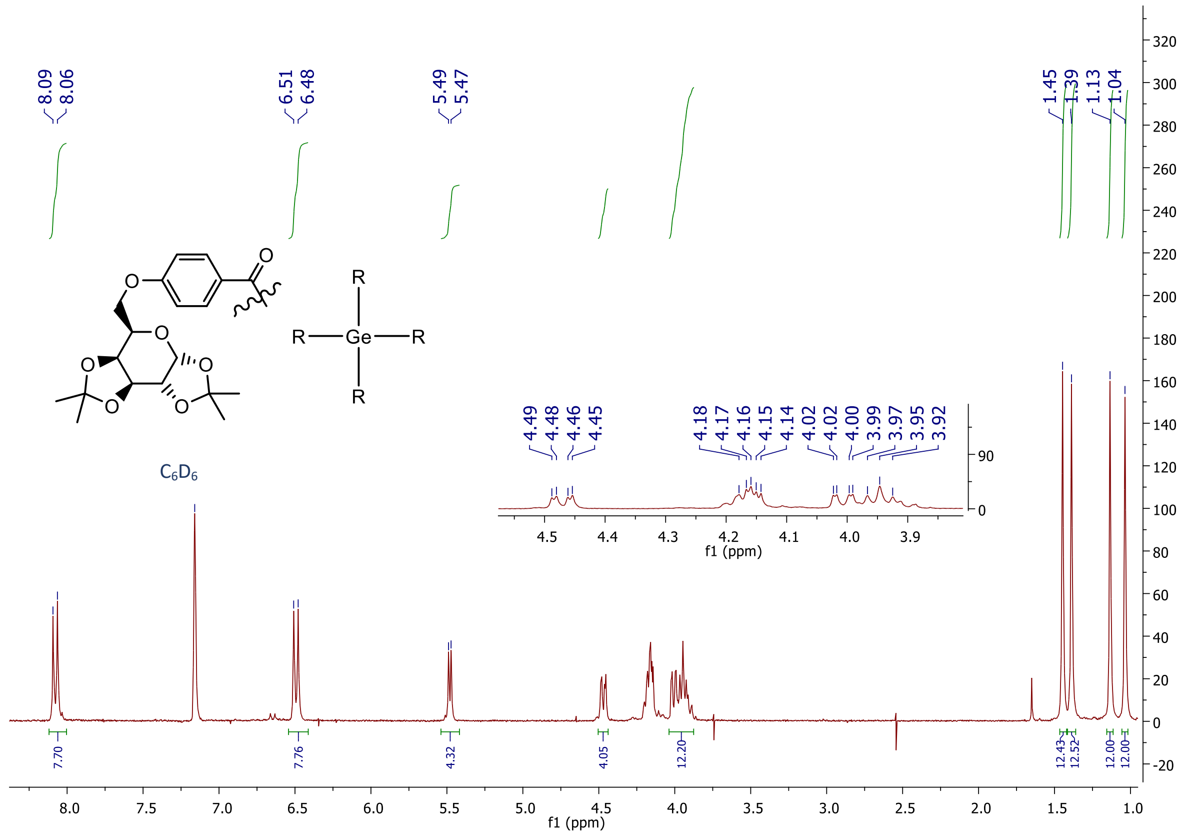

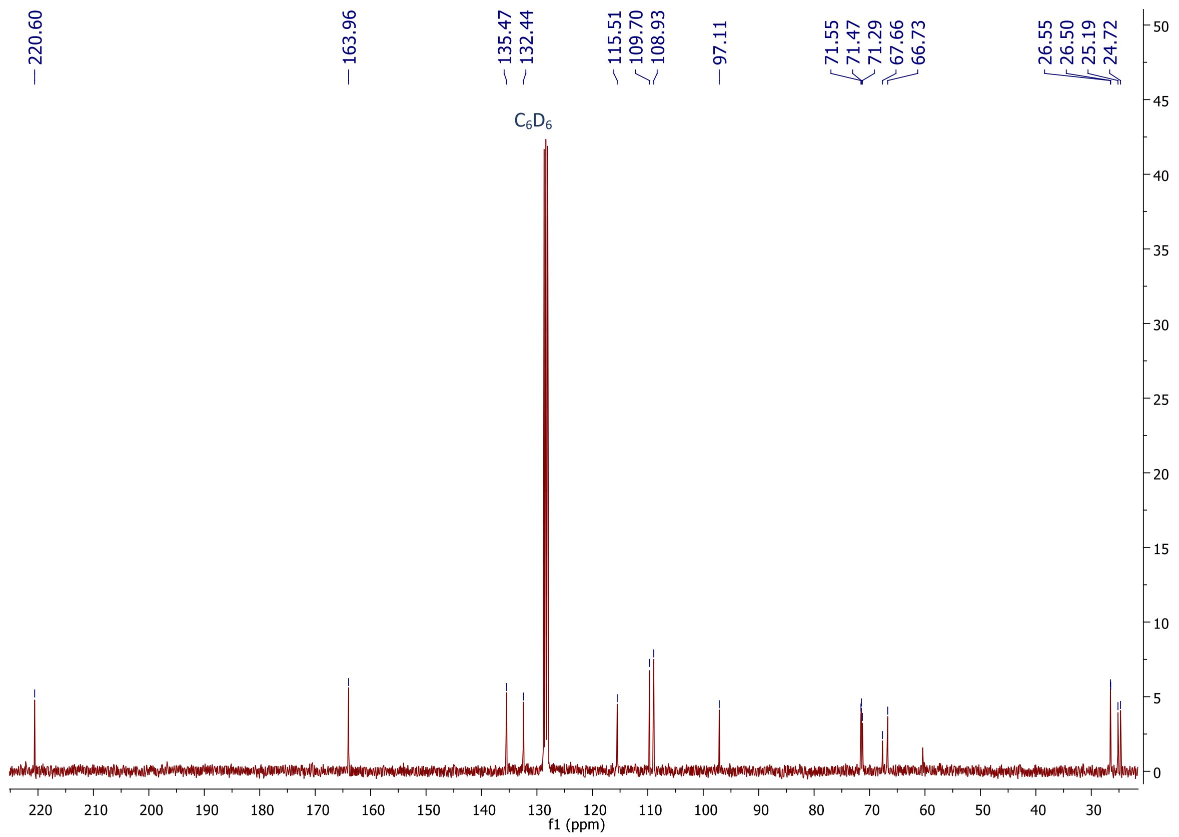

FIgure 6: : ^{1}H-NMR spectra, ^{13}C-NMR spectra of ***7***

3 UV/Vis-Spectroscopy

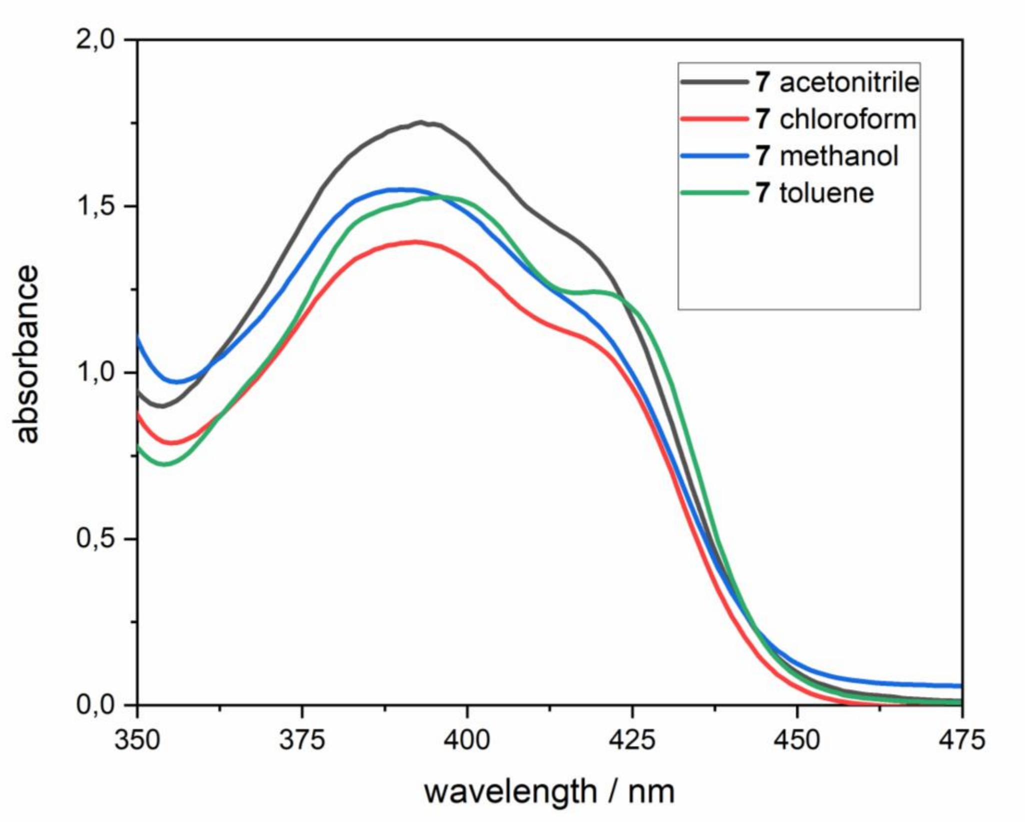

Figure 7: UV/Vis Spectrum of 7 in various solvents

4 Single Crystal X-ray Crystallography

All crystals suitable for single crystal X-ray diffractometry were removed from a vial or a Schlenk and immediately covered with a layer of silicone oil. A single crystal was selected, mounted on a glass rod on a copper pin, and placed in the cold N_2 stream provided by an Oxford Cryosystems cryostream. XRD data collection was performed for compounds **1,2,4** and **6**, on a Bruker APEX II diffractometer[3] with use of an IμS microsource (Incoatec microfocus) sealed tube of Mo Kα radiation (λ= 0.71073 Å) and a CCD area detector. Data integration was carried out using SAINT.[1] Empirical absorption corrections were applied using SADABS.[4-5] The structures were solved with use of the intrinsic phasing option in SHELXT[5] and refined by the full-matrix least-squares procedures in SHELXL[6-10] as implemented in the program SHELXLE.[11] The space group assignments and structural solutions were evaluated using PLATON.[12-14] Non-hydrogen atoms were refined anisotropically. All other hydrogen atoms were located in calculated positions corresponding to standard bond lengths and angles and refined using a riding model. Due to insufficient anomalous dispersion effects, absolute structures were not established in this analysis. However, the absolute configurations of both **1** and **2** were established according to the configuration of the starting materials. Compound **6** was refined as a 2-component inversion twin (BASF 0.03). All crystal structures representations were made with the program Diamond.[15] CIF files were edited, validated and formatted either with the programs encifer,[16] publCIF,[17] or Olex2.[18] CCDC **2041244-2041247** contain the supplementary crystallographic data for compounds **1,2,4** and **6** respectively. These data can be obtained free of charge from The Cambridge Crystallographic Data Centre *via* www.ccdc.cam.ac.uk/data_request/cif. Table **1** contains crystallographic data and details of measurements and refinement for compounds **1,2,4** and **6**. **Table 1**. Crystallographic data and details of measurements for compounds **1,2,4** and **6**
Mo Kα (λ=0.71073Å). $R1= \Sigma/ |F_o|- |F_c|/|\Sigma|F_d$; $wR2 = [\Sigma_w(F_o^2-F_2^2)^2/\Sigma_w(F_o^2)^2]^{1/2}$

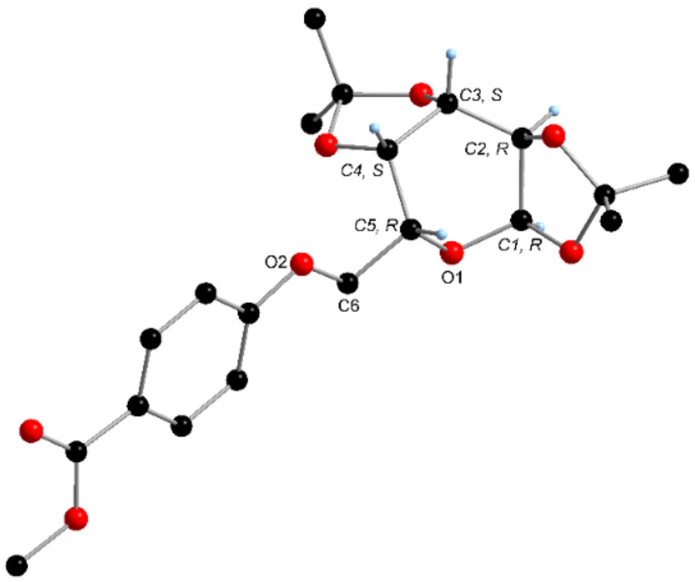

Figure 8: ORTEP representation for compound **4**. Thermal ellipsoids are depicted at the 50% probability level. Hydrogen atoms are omitted for clarity. Selected bond lengths (Å) with estimated standard deviations: O(1)-C(1) 1.407 (2), O(1)-C(5) 1.435 (2), O(2)-C(6) 1.426 (2), O(8)-C(19) 1.209 (3).

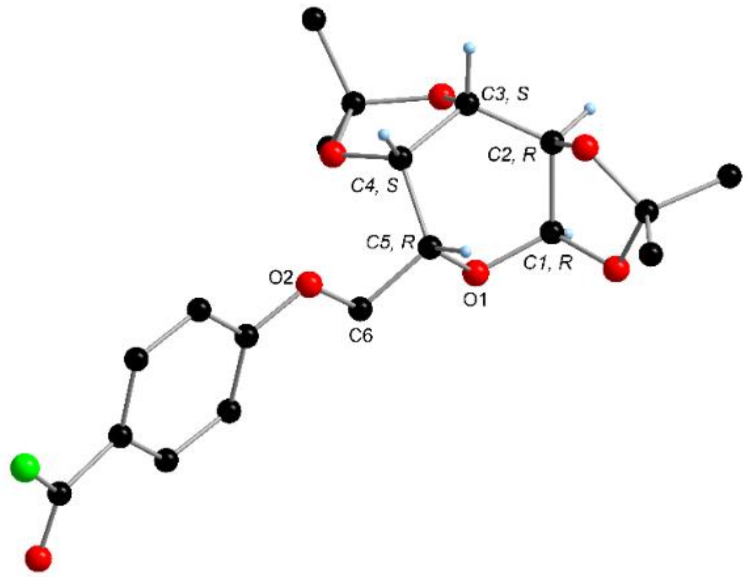

Figure 9: ORTEP Representation for compound **6**. Thermal ellipsoids are depicted at the 50% probability level. Hydrogen atoms are omitted for clarity. Selected bond lengths (Å) with estimated standard deviations: F(1)-C(19) 1.346 (3), O(1)-C(1) 1.410 (3), O(1)-C(5) 1.427 (3), O(2)-C(6) 1.434 (3), O(7)-C(19) 1.198 (3)

Table S1: Crystallographic data and details of measurements for compounds **1,2,4,6**.

**Compound**	**1**<br>CCDC 2041244	**2**<br>CCDC 2041245	**4**<br>CCDC 2041246	**6**<br>CCDC 2041247
Formula	$C_{20}H_{26}O_8$	$C_{19}H_{23}FO_7$	$C_{21}H_{44}O_6Si_4$	$C_{21}H_{44}GeO_6Si_3$
Fw (g mol^{-1})	394.41	382.37	504.92	549.42
a (Å)	12.8475(6)	6.6976(17)	9.9928(5)	9.9756(6)
b (Å)	15.4172(7)	10.441(3)	16.8046(9)	16.7907(9)
c (Å)	15.9975(6)	13.719(4)	17.6187(9)	17.6367(10)
α (°)	90	86.124(9)	90	90
β (°)	110.881(1)	81.359(10)	90	90
γ (°)	90	77.426(10)	90	90
V (Å^3)	2960.6(2)	925.1(4)	2958.6(3)	2954.1(3)
Z	6	2	4	4
Crystal size (mm)	0.10 × 0.09 × 0.08	0.20 × 0.19 × 0.09	0.14 × 0.08 × 0.07	0.15 × 0.10 × 0.09
Crystal habit	Block, colourless	Block, colourless	Block, colourless	Block, colourless
Crystal system	Monoclinic	Triclinic	Orthorhombic	Orthorhombic
Space group	$P2_1$	*P1*	$P2_12_12_1$	$P2_12_12_1$
d_{calc} (Mg m^{-3})	1.327	1.373	1.134	1.235
μ (mm^{-1})	0.10	0.111	0.23	1.19
T (K)	100(2)	100(2)	100(2)	100(2)
2θ range (°)	2.2–28.1	2.5–32.7	2.3–25.8	2.3–32.1
F(000)	1260	404	1096	1168
T_{min}, T_{max}	0.694, 0.747	0.622, 0.747	0.447, 0.747	0.454, 0.747
R_{int}	0.077	0.054	0.146	0.082
No. of measured, independent and observed [I > 2s(I)] reflections	107252, 22568, 16576	41466, 14062, 10272	47124, 5372, 4232	54352, 11284, 9641
independent reflections	22568	14062	5372	11284
No. of parameters, restraints	772, 1	495, 3	293, 0	294, 0
Δ›$_{max}$, Δ›$_{min}$ (e Å^{-3})	0.34, -0.28	0.39, -0.21	0.36, -0.35	1.79, -1.08
R1, wR2 (all data)	R1 = 0.0769<br>wR2 = 0.0998	R1 = 0.0814<br>wR2 = 0.1081	R1 = 0.0754<br>wR2 = 0.0891	R1 = 0.0550<br>wR2 = 0.1023
R1, wR2 (>2σ)	R1 = 0.0454<br>wR2 = 0.0883	R1 = 0.0493<br>wR2 = 0.0962	R1 = 0.0440<br>wR2 = 0.0809	R1 = 0.0422<br>wR2 = 0.0964

5. References:

1. Pangborn, A. B., Giardello, M. A.; Grubbs, R. H.; Rosen, R. K., Timmers, F. J. *Organometallics* **1996**, 15, 1518.
2. Gee, W. J., Hierold, J., MacLellan, J. G., Andrews, P. C.; Lupton, D. W., Junk, P. C. *Chiral Lanthanoid Dimers Ligated by Carbohydrate-Based Diketonates: Catalytic and Luminescent Properties.* Eur. J. Inorg. Chem. **2011** (25) 3755-3760.
3. Bruker *APEX2 and SAINT*, Bruker AXS Inc.: Madison, Wisconsin, USA, 2012.
4. Blessing, R. H., An empirical correction for absorption anisotropy. *Acta Crystallogr., Sect. A: Found. Adv.* **1995,** *51* (1), 33-38.
5. Sheldrick, G. M. *SADABS, Version 2.10, Siemens Area Detector Correction*, Universität Göttingen, Germany, 2003
6. Sheldrick, G. M., SHELXT - Integrated space-group and crystal-structure determination. *Acta Crystallogr., Sect. A: Found. Adv.* **2015,** *71* (1), 3-8.
7. Sheldrick, G. M., Phase annealing in SHELX-90: direct methods for larger structures. *Acta Crystallogr., Sect. A: Found. Adv.* **1990,** *46* (6), 467-473.
8. Sheldrick, G. M., A short history of SHELX. *Acta Crystallogr., Sect. A: Found. Adv.* **2008,** *64* (1), 112-122.
9. Sheldrick, G. M., Crystal structure refinement with SHELXL. *Acta Crystallogr., Sect. C: Struct. Chem.* **2015,** *71* (1), 3-8.
10. Sheldrick, G. M. *SHELXS97*, Univ. Göttingen, Ger. , 1997.
11. Huebschle, C. B.; Sheldrick, G. M.; Dittrich, B., ShelXle: a Qt graphical user interface for SHELXL. *J. Appl. Crystallogr.* **2011,** *44* (Copyright (C) 2014 American Chemical Society (ACS). All Rights Reserved.), 1281-1284.
12. Spek, A. L., Single-crystal structure validation with the program PLATON. *J. Appl. Crystallogr.* **2003,** *36* (1), 7-13.
13. Spek, A. L., Structure validation in chemical crystallography. *Acta Crystallogr., Sect. D: Biol. Crystallogr.* **2009,** *65* (2), 148-155.
14. Spek, A. L., PLATON SQUEEZE: a tool for the calculation of the disordered solvent contribution to the calculated structure factors. *Acta Crystallogr., Sect. C: Struct. Chem.* **2015,** *71* (1), 9-18.
15. Putz, H.; Brandenburg, K. *Diamond - Crystal and Molecular Structure Visualization*, 3.2i; Crystal Impact: Bonn, Germany.
16. Allen, F. H.; Johnson, O.; Shields, G. P.; Smith, B. R.; Towler, M., CIF applications. XV. enCIFer: a program for viewing, editing and visualizing CIFs. *J. Appl. Crystallogr.* **2004,** *37* (2), 335-338.
17. Westrip, S., publCIF: software for editing, validating and formatting crystallographic information files. *J. Appl. Crystallogr.* **2010,** *43* (4), 920-925.
18. Dolomanov, O. V.; Bourhis, L. J.; Gildea, R. J.; Howard, J. A. K.; Puschmann, H., OLEX2: a complete structure solution, refinement and analysis program. *J. Appl. Crystallogr.* **2009,** *42* (2), 339-341.
